# Thermoplasmonic Effect Enables Indirect ON–OFF Control over the Z‐E Isomerization of Azobenzene‐Based Photoswitch

**DOI:** 10.1002/smll.202404755

**Published:** 2024-09-03

**Authors:** Nina Tarnowicz‐Staniak, Mateusz Staniak, Marta Dudek, Marek Grzelczak, Katarzyna Matczyszyn

**Affiliations:** ^1^ Institute of Advanced Materials Faculty of Chemistry Wrocław University of Science and Technology Wyb. Wyspiańskiego 27 Wrocław 50‐370 Poland; ^2^ Institute of Mathematics University of Wrocław pl. Grunwaldzki 2/4 Wrocław 50‐384 Poland; ^3^ Centro de Física de Materiales (CSIC‐UPV/EHU) Donostia International Physics Center (DIPC) Paseo Manuel de Lardizabal 5 San Sebastian 20018 Spain; ^4^ International Institute for Sustainability with Knotted Chiral Meta Matter (WPI‐SKCM2) Hiroshima University Higashihiroshima 739‐8526 Japan

**Keywords:** cellulose, nanoparticles, photochromes, photocontrol, plasmonics, thermoplasmonics

## Abstract

Proper formulation of systems containing plasmonic and photochromic units, such as gold nanoparticles and azobenzene derivatives, yields materials and interfaces with synergic functionalities. Moreover, gold nanoparticles are known to accelerate the *Z*‐*E* isomerization of azobenzene molecules in the dark. However, very little is known about the light‐driven, plasmon‐assisted *Z*‐*E* isomerization of azobenzene compounds. Additionally, most of the azobenzene‐gold hybrids are prepared with nanoparticles of small, isotropic shapes and azobenzene ligands covalently linked to the surface of nanostructures. Herein, a formulation of an innovative system combining azobenzene derivative, gold nanorods, and cellulose nanofibers is proposed. The system's structural integrity relies on electrostatic interactions among components instead of covalent linkage. Cellulose, a robust scaffold, maintains the material's functionality in water and enables monitoring of the material's plasmonic‐photochromic properties upon irradiation and at elevated temperatures without gold nanorods aggregation. Experimental evidence supported by statistical analysis suggests that the optical properties of plasmonic nanometal enable indirect control over the *Z*‐*E* isomerization of the photochromic component with near‐infrared irradiation by triggering the thermoplasmonic effect. The proposed hybrid material's dual plasmonic‐photochromic functionality, versatility, and ease of processing render a convenient starting point for further advanced azobenzene‐related research and 3D printing of macroscopic light‐responsive structures.

## Graphical abstract

Gold nanoparticles and azobenzene derivatives can be incorporated within one, processable, water‐functional material by using cellulose nanofibers as a functional scaffold. Presence of gold nanorods enables light‐driven, plasmon‐assisted *Z‐E* isomerization of a photochrome. Statistical modelling indicates that thermoplasmonic effect, triggered upon red‐NIR irradiation, grants an ON–OFF control over the isomerization kinetics in the hybrid material.

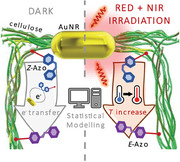

## 1 Introduction

The isomerization of azobenzene and its derivatives (Azo) has remained topical since the 1930s.^[^ ^1^ ^]^ Deployment of Azo photoswitches^[^ ^2^ ^]^ requires proper harnessing of their collective activity^[^ ^3^, ^4^, ^5^ ^]^ and control over their properties by adjacent chemical species or antagonistic external energy sources (e.g., light and temperature).^[^ ^6^, ^7^, ^8^ ^]^ Traditionally, metallic gold has been widely used to immobilize amine‐ and thiol‐terminated Azo molecules either on planar macroscopic surfaces^[^ ^9^, ^10^, ^11^, ^12^ ^]^ or on positively curved surfaces, such as gold nanoparticles (AuNPs).^[^ ^13^, ^14^, ^15^, ^16^, ^17^, ^18^ ^]^ Current research is mainly devoted to hybrids formed via covalent linkage between the photochromic unit and the nanoplasmonic core (Azo‐AuNPs), and substantial emphasis is put on the investigation of the Light‐Induced Self‐Assembly (LISA) phenomenon.^[^ ^8^, ^15^, ^16^, ^18^ ^]^ The radial distribution of Azo on AuNPs suffers from the need of using small, spherical nanostructures^[^ ^13^, ^18^, ^19^ ^]^ that exhibit limited light‐harvesting properties. Also both inter‐ and intraband transitions in spherical AuNPs overlap with the absorption maxima of the π→π^*^ and n→π^*^ transitions of Azo compounds, thus greatly limiting exploration of potential plasmon‐assisted Azo isomerization. On top of that, the properties of Azo‐AuNPs hybrids are mainly investigated in organic solvents,^[^ ^20^, ^21^ ^]^ yet water‐functional plasmonic‐photochromic systems could be beneficial for biological and medical applications.^[^ ^22^ ^]^ These limitations can be overcome by exploring novel composite formulations where Azo and AuNPs are incorporated in a matrix or scaffold, such as poly(vinyl alcohol),^[^ ^23^ ^]^ polydopamine,^[^ ^24^, ^25^ ^]^ carbon nanotubes,^[^ ^26^ ^]^ or even DNA.^[^ ^27^ ^]^ We perceive that the introduction of a third, inert component can expand the palette of available plasmonic‐photochromic systems, as well as lead to the incorporation of entirely new classes of Azo molecules and bigger nanoplasmonic cores of anisotropic geometries. As a result, this approach can give rise to new, previously unattainable possibilities and experimental scenarios.

Furthermore, new Azo‐AuNPs formulations can contribute to the ongoing research on the modulation of Azo isomerization in terms of the expansion of Azo excitation wavelengths into the red or near‐infrared (NIR) range^[^ ^6^, ^7^, ^28^ ^]^ or modification of the *Z*‐isomers lifetimes.^[^ ^29^, ^30^ ^]^ These goals can be achieved not only by synthetic modification of Azo molecules^[^ ^31^, ^32^, ^33^, ^34^, ^35^ ^]^ but also by alternative strategies (often called indirect modifications) relying on electron transfer^[^ ^6^, ^36^, ^37^, ^38^ ^]^ or intra‐ and intermolecular energy transfer.^[^ ^39^, ^40^, ^41^, ^42^, ^43^, ^44^, ^45^, ^46^, ^47^ ^]^ Alternative approaches are especially appealing for multi‐component systems, in which the properties of the already existing Azo molecules are modified and broadened beyond their intrinsic limitations, while the profitable features, such as robustness, are maintained.^[^ ^6^, ^45^ ^]^

For a long time, AuNPs have been mentioned in the context of indirect modification of Azo photoswitching as multiphoton sensitizers.^[^ ^48^ ^]^ Recently, however, AuNPs have been recognized^[^ ^37^, ^38^ ^]^ as factors modulating Azo *Z‐E* isomerization in the dark via electron transfer (eT), as proposed for the first time by Hallett‐Tapley et al.,^[^ ^49^ ^]^ and further supported in the ensuing research.^[^ ^50^, ^51^, ^52^ ^]^ However, these reports focused solely on the catalytic influence of spherical nanoparticles.

Shape‐limitation of plasmonic cores is understandable due to the possible aggregation of bigger and anisotropic nanostructures in the presence of Azo, especially in organic solvents. Nonetheless, by using small, isotropic nanoparticles exhibiting limited light‐harvesting properties, studies were restricted to the exclusive investigation of Azo's dark (thermal) *Z*‐*E* isomerization. Yet, plasmonic nanostructures are a source of powerful light‐induced catalytic tools^[^ ^53^, ^54^ ^]^ such as heat,^[^ ^55^ ^]^ electric field enhancement,^[^ ^56^ ^]^ and hot (i.e., highly energetic) carriers.^[^ ^57^ ^]^ It can be hypothesized that these phenomena could be potentially used to indirectly control Azo isomerization, both *E*‐*Z* and *Z*‐*E* reactions, affecting their mechanisms and kinetics.

**Figure 1:**
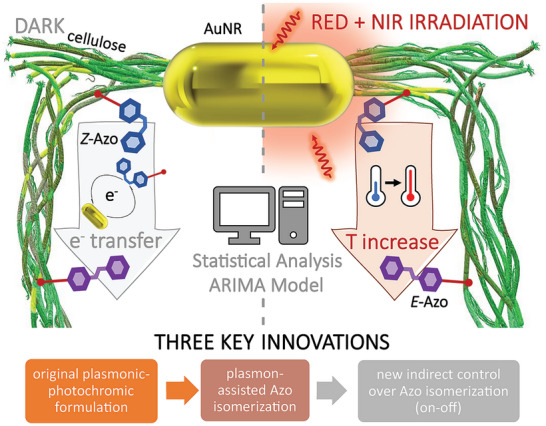
Cellulose nanofibers provide simultaneous support for two independent functional units in the hybrid material, namely plasmonic (gold nanorod, AuNR) and photochromic (azobenzene derivative, Azo) components, preserving their optical properties and preventing aggregation in water (innovation 1). Presence of AuNRs enables performance of plasmon‐assisted Azo isomerization under red‐NIR irradiation (innovation 2). Under the light conditions, which are not accessed by the photochrome, AuNRs convert absorbed energy into heat, resulting in a faster Azo *Z‐E* isomerization, as revealed upon statistical modelling of the kinetic data. Statistical analysis also reveals that switching between dark and light conditions grants an instantaneous, indirect ON–OFF control over the photochromic reaction in the hybrid material via the thermoplasmonic effect (innovation 3).

We propose an innovative, light‐addressable macroscopic plasmonic‐photochromic material comprising cellulose nanofibers (CNFs), gold nanorods (AuNRs), and Azo molecules (AzoGly), in which the control over the kinetics of the Azo isomerization reaction is granted via the thermoplasmonic effect (**Figure** **1**). Due to the use of CNFs as a scaffold for both functional components, the proposed hybrid material exhibits stable plasmonic and photochromic properties and structural integrity in water under elevated temperatures and UV–vis–NIR irradiation. The indirect control over Azo *Z‐E* isomerization was achieved due to the proper material design excluding the spectral overlapping of metallic and molecular components and enabling efficient triggering of the thermoplasmonic effect upon irradiation in the red‐NIR spectral range. Hence, the proposed plasmon‐assisted process constitutes a new pathway of indirect modulation of Azo photochromism. We also propose means to quantify the extent of the thermoplasmonic effect by monitoring kinetic changes in the Azo *Z‐E* isomerization, thus indicating the prospects for Azo molecules as molecular thermometers. Finally, through advanced statistical modelling (Autoregressive Integrated Moving Average, ARIMA^[^ ^58^ ^]^ model) of the kinetic data, we confirm the major contribution of the thermoplasmonic effect upon irradiation and the instantaneous ON–OFF control it grants over Azo *Z‐E* isomerization.

## 2 Results and Discussion

**Figure 2:**
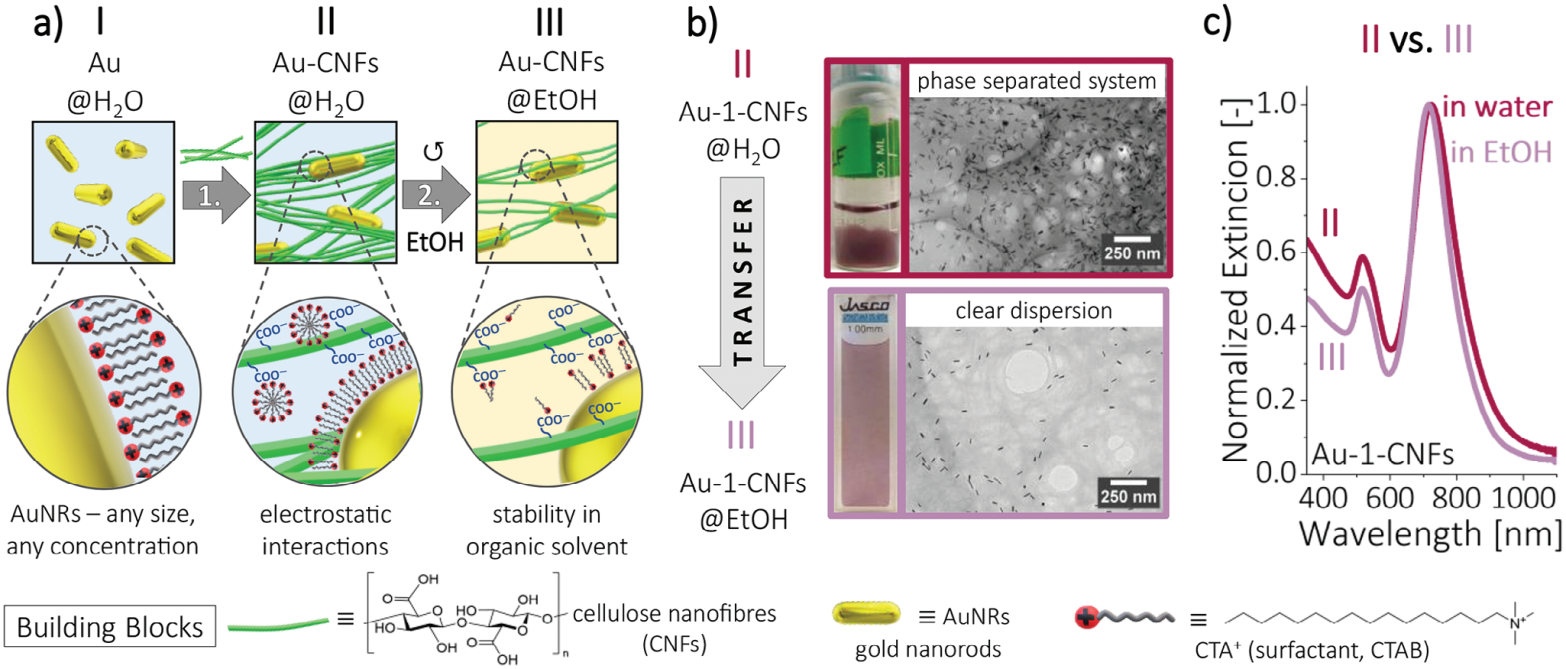
Preparation of Au‐CNFs pre‐composite. a) Schematic representation of the underlying interactions between components. b) TEM images of Au‐1‐CNFs in water and ethanol (EtOH) present the material's structural integrity and its ability to form self‐standing films upon drying. Insets: digital images of the samples showing differences in the physical appearance of the pre‐composite before and after transfer to EtOH. c) Normalized UV–vis–NIR extinction spectra of the water and EtOH dispersions of Au‐CNFs pre‐composite. Colour codes between panels (b and c) are matching.

To investigate plasmon‐assisted isomerization of azobenzene derivative, we designed formulation containing azobenzene modified with glycine (AzoGly, see Scheme S1 and Figure S3, Supporting Information), gold nanorods (AuNRs, Figures S4 and S5, Supporting Information), and cellulose nanofibers (CNFs, TEMPO, 2,2,6,6‐tetramethylpiperidinyl‐1‐oxyl, oxidized) as a scaffold. Such design allows to preserve the intrinsic optical properties of both functional components and ensures their compatibility and functionality in water. In the first step, we prepared pre‐composite material by relying on electrostatic interactions between CTAB‐coated AuNRs and CNFs.^[^ ^59^, ^60^, ^61^, ^62^, ^63^, ^64^, ^65^ ^]^ Nanoparticles, stabilized by a positively charged surfactant (cetyltrimethylammonium bromide, CTAB), containing $-N{\left(CH_{3}\right)}_{3}^{+}$ head group, (**Figure** **2**a – stage I), were uniformly immobilized on a cellulose scaffold rich in carboxyl groups, − *COO* ^−^ (Figure 2a – stage II). The adsorption of CTAB (as micelles or bilayer on AuNRs) on cellulose lowered the hydrophilicity of the fibers^[^ ^59^, ^66^, ^67^ ^]^ and resulted in the phase separation of Au‐decorated CNFs, facilitating further post‐processing of the fibers (Figure 2b, top).

To enable the incorporation of water‐insoluble AzoGly (Figure S6, Supporting Information) the pre‐composite was transferred to ethanol (EtOH) forming a clear dispersion (Figure 2a – stage III and Figure 2b). Typically, CTAB‐stabilized nanoparticles aggregate in pure EtOH, due to the surfactant shell destruction as a result of the 250 times higher critical micelle concentration of CTAB in EtOH compared to water. However here, cellulose scaffold replaced, to some extent, CTAB in its protective role and ensured the stability of AuNRs, preserving the material's structural integrity (Figure 2b) and optical properties (Figure 2c). Thus, our easy to replicate approach enables stabilization of the optical properties of the plasmonic component in EtOH, regardless of size or shape of AuNPs.

**Figure 3:**
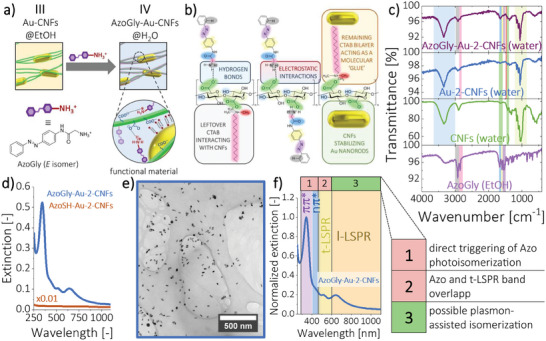
AzoGly‐Au‐2‐CNFs – hybrid plasmonic‐photochromic fibers. a) Impregnation process and schematic depiction of the AzoGly‐CNFs interactions within the material's structure. b) Proposed chemical structure with a visual indication of the underlying interactions. Color codes match highlights on the IR spectra to facilitate the analysis. c) IR spectra of the samples in solid state reveal the role of the underlying electrostatic interactions and hydrogen bonds in providing material's structural integrity. d) Typical sulphur‐containing ligands exhibit worse affinity to Au‐CNFs framework compared to AzoGly. The resulting materials aggregate, and their photochromic and plasmonic properties are compromised. e) TEM image of the AzoGly‐Au‐2‐CNFs sample, indicating a homogenous distribution of components. f) UV–vis–NIR spectrum of the hybrid sample, showing three spectral regions for potential use for 1) direct photoisomerization of the Azo component, 2) simultaneous triggering of the photoisomerization and plasmon‐related effects (not used in this work), 3) exclusive triggering of the plasmon‐derived effects and gaining potential indirect photocontrol over Azo isomerization.

The as‐prepared Au‐CNFs pre‐composite dispersed in EtOH served as a platform for impregnation with the photochromic dye of choice via dye sorption during an overnight incubation step. We used a model photochrome, AzoGly (Figure S3, Supporting Information), in the form of salt to promote interactions between its protonated end group, $-NH_{3}^{+}$, and − *COO* ^−^ groups of CNFs (**Figure** **3**a – stage IV). CNFs are rich in − *COO* ^−^ groups and, hence, trifluoroacetate counterion (TFA^–^) of azobenzene salt does not remain in the composite. The resulting plasmonic‐photochromic material (Figure 3b) was transferred back to water, and the presence of the photochromic unit in the final formulation was confirmed using IR spectroscopy (Figure 3c; Figure S7 and Table S1, Supporting Information, IR spectra were measured for samples evaporated under vacuum), a typical technique used for the characterization of cellulose‐based materials.^[^ ^68^, ^69^, ^70^ ^]^

The integrity of the composite was assured by intermolecular interactions (electrostatic attraction and hydrogen bonds formation, Figure 3b) between AzoGly and CNFs. A peak appearing at 2924 cm^−1^ for AzoGly (− *N* − *H* stretching of $-NH_{3}^{+}$ end group, grey highlight) was shifted to 2918 cm^−1^ in AzoGly‐Au‐2‐CNFs. Simultaneously, the position of the symmetrical vibration band of − *COO* ^−^ group of CNFs (vivid green highlight) shifted from 1429 cm^−1^ in Au‐2‐CNFs to 1419 cm^−1^ in the final hybrid material. Moreover, the reasonable assumption of CTAB's role of a molecular glue in the final formulation was confirmed by analyzing the position of − *CH*_3_ band of ammonium salt appearing at 1456 cm^−1^ (vivid red highlight, Figure S8, Supporting Information). Position of this band was established in the literature to identify presence of CTAB in the cellulose‐surfactant materials.^[^ ^68^, ^69^ ^]^ Its presence indicates that the AzoGly‐Au‐2‐CNFs composite contains residual CTAB molecules. Because a thorough washing process excludes free surfactant molecules in bulk solution, it is feasible to assume that CTAB remains intercalated in the system either 1) solely on the surface of CNFs or 2) as a residual bilayer with lower integrity, mediating Au‐CNFs interactions (Figure 3b).

Other peaks in the AzoGly‐Au‐2‐CNFs sample were also identified (Table S1, Supporting Information) and confirmed material composition and its structural integrity: *C*—*C* cellulose ring stretching at 1161 cm^−1^, *C*—*O*—*C* cellulose pyranose ring stretching centred at 1059 cm^−1^, and *C*—*O*—*C* cellulose *β*‐glycosidic linkages at 905 cm^−1^ (all marked with pale yellow color), and AzoGly bands occurring as a part of the broad 1653 cm^−1^ peak, namely —*C*=*O* amide signal (marked with vivid green), *C*=*C* (in plane, bright yellow), and *N*=*N* (pale purple). Finally, we postulate that gold nanorods have negligible role in mediating AzoGly‐CNFs interactions. Impregnation of bare CNFs with AzoGly yielded composites exhibiting photochromic bands, thus indicating high efficiency of dye adsorption on the fibers (Figure S9, Supporting Information). In principle, the higher the concentration of AzoGly during impregnation, the better the photochromic properties of the resulting composite. The increase of ≈3.8 times in dye concentration gave ≈26 times higher intensity of the π→π^*^ band (based on the corrected values in Figure S9b, Supporting Information; comparison between concentrations 0.69 and 2.62 mm), emphasizing profits from impregnations performed at higher AzoGly concentrations. These results are pivotal and emphasize the strength and importance of intermolecular interactions in the designed material (Figure 3b).

Use of CNFs is central to our material design, since it excludes the need for covalent anchoring of dye molecules on the surface of Au through the Au─S bond, which often hinders photochromism of the dyes, due to the steric hindrance imposed by metal surface. To demonstrate that thiol‐terminated Azo underperform AzoGly, we used thiolated ligands that have already been employed to form self‐assembled monolayers (SAMs) on metallic gold,^[^ ^13^, ^71^ ^]^ namely THF‐soluble AzoSH (Figure S10a, Supporting Information) and EtOH‐soluble AzoSS (Figure S10b, Supporting Information). The as‐prepared materials lacked plasmonic and photochromic properties (Figure 3d; Figure S11 and Section 3.3, Supporting Information). Hydrophobic AzoSH and AzoSS prevent hybrids from dispersing in water, causing aggregation, whereas more polar, protonated AzoGly formed with Au‐CNFs the water‐functional plasmonic‐photochromic material. Hence, our protocol constitutes an important contribution to the already existing knowledge.

Typically, transfer of water‐insoluble photochrome to the aqueous environment results in its aggregation, as presented by Sun and co‐workers, who used nanocrystalline cellulose as a platform for hydrophobic spirooxazine derivative.^[^ ^72^ ^]^ Our material does not contain any clumps of Azo, as confirmed by transmission electron microscopy (TEM, Figure 3e). Our protocol enables uniform impregnation of nanofibers and assures homogenous nature of the hybrid material. CNFs ensure system's stability, and the as‐prepared AzoGly‐Au‐2‐CNFs composite maintains its dual plasmonic‐photochromic signatures, as shown on the UV–vis–NIR absorption spectra (Figure 3f). The intensities of the π→π^*^ and n→π^*^ photochromic bands in a solvent in which AzoGly is not soluble clearly confirm the success of the proposed material design in transferring Azo properties to the new environment. We attribute small differences in their positions and profiles compared with pristine AzoGly (Figure S3, Supporting Information) to the solvent change^[^ ^73^, ^74^ ^]^ and interactions with CNFs.^[^ ^72^ ^]^ Localized Surface Plasmon Resonance (LSPR) bands of Au‐2 (transverse, t‐LSPR and longitudinal, l‐LSPR) are distinct, well separated, and not distorted. Slight spectral shifts, namely 3 nm blueshift and 18 nm redshift for t‐LSPR and l‐LSPR, respectively (Figure S12, Supporting Information), can be attributed to the change in the effective refractive index due to the CTAB exchange at the surface of AuNRs.^[^ ^75^ ^]^ In principle, we distinguish three different spectral regions of the sample as marked on top of Figure 3f. The first (1) covers the π→π^*^ and part of the n→π^*^ band of AzoGly. This spectral region can be used to directly trigger photoisomerization of the photochromic component. The second (2) corresponds with partially overlapping Azo n→π^*^ band and t‐LSPR band of Au‐2. AzoGly undergoes *Z*‐*E* isomerization when irradiated in a close proximity to the n→π^*^ band, e.g. 500–600 nm (Figure S13, Supporting Information). This precludes us from further use of region (2) in the plasmon‐assisted experiments. Otherwise, it would be impossible to assess which phenomenon (direct Azo isomerization or plasmon‐related effects) is responsible for the potential photocontrol of the occurring reaction. The third region (3), covers the position of the l‐LPSR band of Au‐2 and is the most suitable to gain potential indirect control over Azo isomerization process via plasmon‐derived phenomena. In summary, our approach enables efficient preparation of stable, hybrid materials with well‐defined optical characteristics and maintained plasmonic and photochromic signatures. Incorporation of anisotropic AuNPs enables beneficial separation of the components spectral features, which can be exploited to perform plasmon‐assisted isomerization of the photochrome.

**Figure 4:**
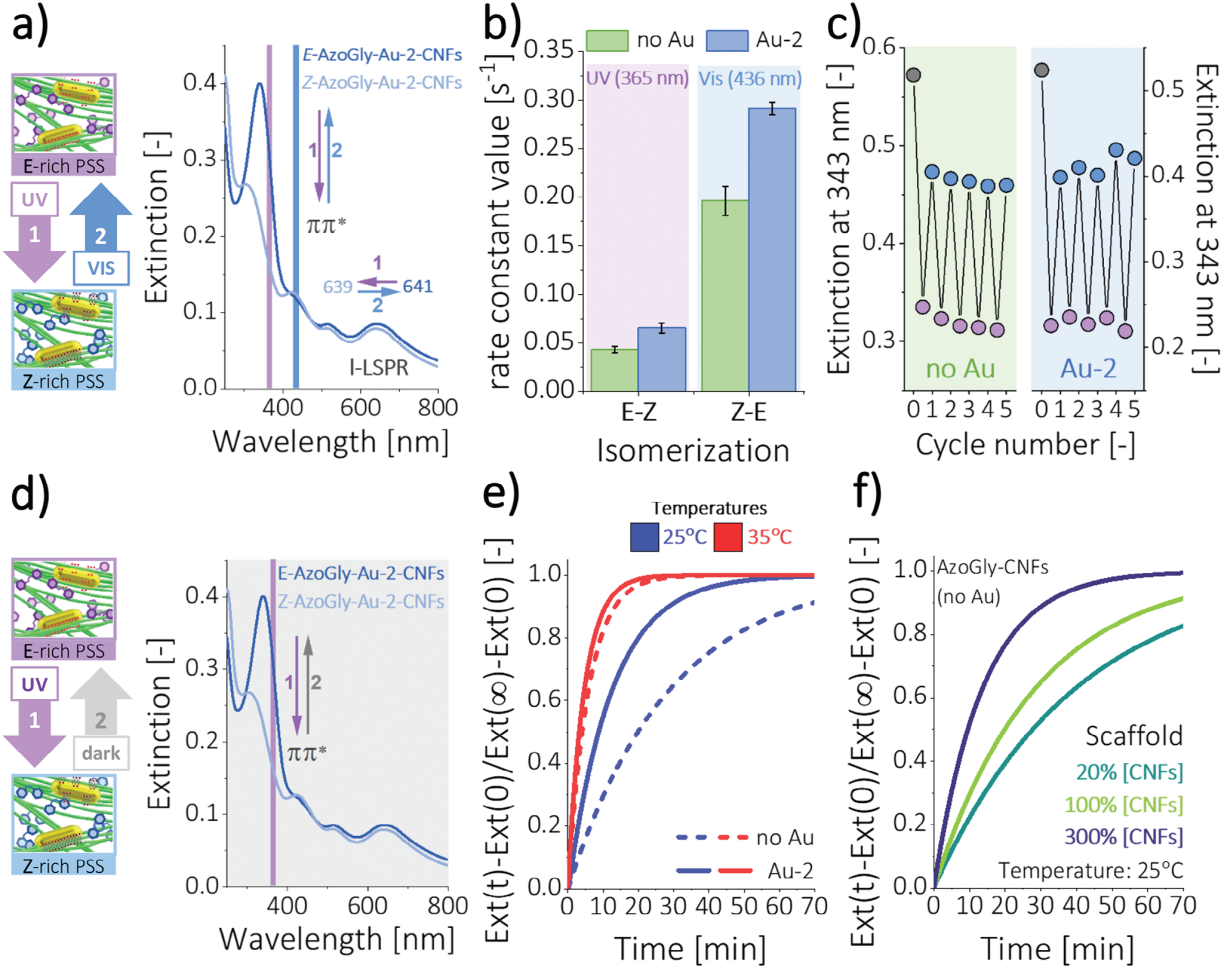
Photochromism, photostability, and thermal relaxations of hybrid samples. a) Azo‐component maintains photochromic functionality in the hybrid material and undergoes *E‐Z* and *Z‐E* isomerization upon UV and visible light irradiation, respectively. Plasmonic properties of the material are preserved the whole time. b) Presence of AuNRs grants a kinetic boost of *E‐Z* (+52.2%) and *Z‐E* (+48.6%) isomerizations compared to control sample without Au. c) Hybrid materials exhibit distinct and stable photoswitching upon interval UV (3 min) and visible light (3 min) irradiation throughout many cycles. π→π^*^ band intensities are indicated by purple points at PSS_Z_ and blue points at PSS_E_. d) Hybrid samples exhibit thermal *Z‐E* isomerization in the dark, just as pristine azobenzenes. e) Thermal back‐relaxation in the dark is faster in the presence of gold. Kinetic boost decreases with increasing temperature, due to the difference in the activation energy of the process in the presence of AuNRs. f) CNFs influence the isomerization of AzoGly by creating specific environmental conditions. The CNFs network imposes steric constraints and confinement, hence changing the stability of the photochrome. With increasing CNFs content, Azo thermal back‐isomerization occurs faster.

The main functionalities of any Azo‐containing material are its photoswitchability, photo‐, and thermostability (**Figure** **4**). To evaluate the photochromic behavior, we subjected AzoGly‐Au‐2‐CNFs hybrid sample to the alternate induction of the *E*‐*Z* and *Z*‐*E* isomerization of the photochromic component (Figure 4a, left). The drop of the π→π^*^ band upon UV (365 nm) irradiation and its rise upon irradiation at 436 nm (Figure S14, Supporting Information) indicated the occurrence of the *E*‐*Z* and *Z*‐*E* isomerizations, respectively (Figure 4a, right). The observed changes are coherent with photoswitching of pristine AzoGly (Figure S3, Supporting Information) and AzoGly‐CNFs control sample (Figure S15c,d, Supporting Information). Importantly, the optical properties of plasmonic component were preserved during the cycle of *E‐Z* and *Z‐*E isomerizations. Only a slight, reversible shift of 2 nm in the position of l‐LSPR band was observed, without any significant distortions or damping (Figure 4a). This is a key difference between our material and other literature Azo‐plasmonic hybrids, where *E‐Z* isomerization causes nanoparticles aggregation. In our design, due to the macroscopic size of CNFs, the collective molecular motion of AzoGly with respect to the plane of the *N*  =  *N* bond is not causative enough to result in aggregation or global changes in the material's 3D structure.

The AuNRs content in the hybrid formulation was adjusted to provide distinct plasmonic contribution to the overall optical properties of the composite, not to provide catalytic or stoichiometric amount of Au for the Azo isomerization process. Nevertheless, the catalytic effect of AuNRs, meaning their favorable influence on the Azo isomerization kinetics and thermodynamics, can be observed when reaction rate constants for hybrid materials with (Au‐2) and without Au (no Au) are compared. Analysis of the extinction changes at the maximum of the π→π^*^ band plotted as a function of time reveals that both reactions obey first‐order kinetics (Figure S15, Supporting Information), just as free Azo molecules in solution. *E*‐*Z* and *Z*‐*E* AzoGly isomerization reactions in the presence of gold are enhanced by 52.2% and 48.6%, respectively (Figure 4b; Figure S15, Supporting Information). These changes reflect the catalytic influence of AuNRs on directly photoinduced Azo isomerization. We postulate that the catalytic boost occurs via the electron transfer (eT) mechanism, as it has been proposed for Azo photoswitching in the dark in the presence of Au nanospheres.^[^ ^49^ ^]^ We hypothesize that eT is triggered by UV irradiation addressing interband transitions of AuNRs, which gives rise to the generation of highly energetic carriers. Typically, the electron transfer from and to AuNRs can be confirmed by changes in the position of the l‐LSPR band.^[^ ^76^ ^]^ Such coherent and reversible changes of the l‐LSPR position occurring during Azo photoisomerization in the hybrid material are depicted in Figure 4a and Figure S16b (Supporting Information).

Both types of hybrid materials (with and without Au) exhibit stable photoswitchability upon interval UV (3 min) and visible light (3 min) irradiation (Figure 4c; Figure S16, Supporting Information). *E‐Z* and *Z‐E* isomerizations can be induced and repeated in the materials over many cycles. The intensity of the π→π^*^ band in both photostationary states remains stable over the course of the experiment. This proves that the degradation of photochromic component or material does not occur.

Azobenzenes, as T‐type photochromes, exhibit faster *Z*‐*E* isomerization in the dark with the increasing temperature. We measured thermal back‐isomerizations of AzoGly component at several temperatures to assess kinetic and thermodynamic differences between the stability of the *Z*‐isomer in materials with and without Au. Samples were first irradiated with UV (to reach *Z*‐rich photostationary state, PSS*_Z_*), and then, their return at a specific temperature in the dark to the *E*‐rich PSS (Figure 4d) was monitored by measuring changes in the extinction of the π→π^*^ AzoGly band as a function of time. Collected data confirm the first‐order kinetics of the process (Figure S17, Supporting Information). We determined the set of kinetic and thermodynamic parameters of the ongoing reaction (Table S2, Supporting Information) by employing Arrhenius (Equation S2, Supporting Information) and Eyring (Equation S3, Supporting Information) equations. In the presence of AuNRs, the activation energy of the process is ≈30 kJ mol^−1^ lower than for the control sample without gold (Table S2, Supporting Information), proving catalytic role of AuNRs in the dark. Notably, catalytic contribution of AuNRs remains clear also at elevated temperatures, since sample with Au‐2 plasmonic core reaches a plateau at 35 °C faster than the composite without gold (Figure 4e). AuNPs are known to catalyze Azo isomerization in the dark via the electron transfer.^[^ ^49^, ^50^, ^52^ ^]^ Since eT can be confirmed based on the redshift of the l‐LSPR band of AuNRs,^[^ ^76^ ^]^ the observed gradual spectral change (ca. 1.8 nm redshift) during Azo *Z‐E* isomerization in the dark confirms electron transfer from AuNRs to Azo (Figure S18, Supporting Information).

Conditions, such as solvent type,^[^ ^74^ ^]^ presence of other chemical species,^[^ ^8^ ^]^ or environmental constraints,^[^ ^77^ ^]^ are known to affect isomerization of Azo. Hence, we investigated influence of bare CNFs scaffold and observed substantial acceleration of the thermal back‐isomerization when molecules are distributed within the CNFs matrix. By transferring AzoGly to AzoGly‐CNFs (water), one creates conditions in which thermal half‐life of the *Z*‐isomer at 25 °C drops from 24.6 h (free AzoGly in EtOH, Figure S19, Supporting Information) to 19.8 min. We hypothesize that this dramatic decrease originates from two effects, namely solvent change and Azo entrapment in the material's structure. To evaluate the latter contribution we tracked thermal isomerization at 25 °C for materials containing different amounts of CNFs and no AuNRs. Since Azo exhibits first‐order kinetics of thermal isomerization, the rate constant is neither dependent on molecule's concentration in the hybrid material nor on material content in the dispersion. The threefold increase of CNFs content (300%[CNFs]) results in the decrease of the thermal half‐life to 9.5 min, while the fivefold decrease of CNFs content (20%[CNFs]) results in the increase of *τ_1/2_* to 27.7 min (Figure 4f; Figure S20, Supporting Information). Since one can rule out the catalytic effect of CNFs,^[^ ^59^ ^]^ the role of cellulose relies on creation of specific material and environmental conditions. Because AzoGly is tethered to CNFs via a glycine‐like side group, its movement and steric freedom are limited. At the same time, CNFs form an entangled network and its density is controlled by mutual interactions between the fibers. At high CNFs concentration fibers interactions are more probable, thus contributing to the steric hindrance and confinement experienced by Azo molecules. Hence, at high CNFs concentration Azo isomerization occurs faster. Our findings are in line with the previously presented results indicating decreased azobenzene switchability due to the loss of conformational freedom.^[^ ^77^ ^]^ It is also possible that interactions between the hydroxyl groups of CNFs and *N* = *N* Azo bond, increased at high CNFs content, contribute to the dramatic decrease of the *τ*_1/2_, similarly to Azo isomerization in the presence of background ligands.^[^ ^8^ ^]^ Notably, the confinement imposed on AzoGly in this system is not too rigid, as AzoGly molecules maintain their overall photoswitchability (Figure 4c).

Presented thermal relaxations of AzoGly‐Au‐2‐CNFs constitute valid points for further development of hybrid materials with desired stabilities of photochromic components. We identified key points of the material's design that influence its final photochromic functionality. We also determined for the first time a complete set of thermodynamic parameters describing thermal back‐isomerization of Azo in the presence of gold and presented that standard Azo‐related research procedures can be applied to investigate complex hybrid systems.

**Figure 5:**
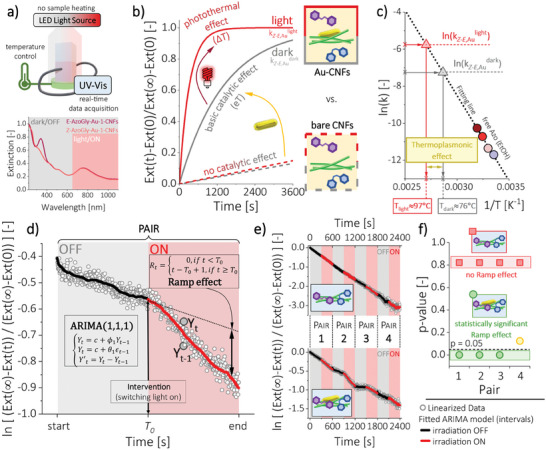
Plasmon‐assisted isomerization of AzoGly a–c) as a free molecule in EtOH and in the presence of Au‐1‐CNFs pre‐composite or d–f) in the hybrid AzoGly‐Au‐1‐CNFs material in water (19.6 wt.% of Au with respect to the mass of cellulose in both scenarios). a) Measurements were performed using custom experimental setup with real‐time data acquisition. Spectral range of irradiation (650–1100 nm) matched the position of the l‐LSPR band of AuNRs and did not trigger the isomerization of AzoGly. b) Bare CNFs scaffold is inert and does not influence the isomerization of AzoGly. The catalytic effect of AuNRs manifests upon the addition of pre‐composite in the dark due to the electron transfer mechanism. Upon irradiation thermoplasmonic effect boosts thermal back‐isomerization of AzoGly by two orders of magnitude. c) Rate constants describing AzoGly *Z‐E* isomerization in the presence of Au can be recalculated into virtual temperatures of the process, and hence, the extent of the thermoplasmonic effect (21 °C) can be estimated. d) Data collection for hybrid materials in water requires a special experimental design (OFF–ON interval experiment) and the use of statistical modelling for data analysis. Autoregressive Integrated Moving Average (ARIMA)‐based approach was used. Dependent variable Y_t_ is forecast based on the previous observations (Y_t‐1_), random error term (ϵ_t‐1_), and stability of the time series (Y’_t_). Illustration depicts ARIMA (1,1,1) and Ramp effect estimation as a method to evaluate influence of switching light on (the intervention). Influence of light is manifested in the slope change of the data subseries (paired dark and light intervals). e) Presence of AuNRs (bottom) grants photocontrol over Azo isomerization in an ON–OFF manner, which manifests as a characteristic breaking of the linear trend of the data at times of the intervention. Irradiation does not affect the reaction when AuNRs are not present in the hybrid sample. f) Quantitative measure of statistical significance of the calculated Ramp effect estimates depicted by comparing p‐values of the significance tests for each modelled pair between Au‐containing sample and control sample without Au. Results indicate that only in the presence of Au the change in the slope of the data upon irradiation is statistically significant. Hence, plasmon‐assisted isomerization of AzoGly in the hybrid material via triggering of the thermoplasmonic effect is entirely manifested.

To show plasmon‐assisted isomerization of Azo and the possibility of an instantaneous indirect photocontrol of this reaction in an ON–OFF manner, we designed proof of concept experiments. We employed crucial advantage of our hybrid material, which is presence of big, anisotropic nanostructures. We implemented gold nanorods (Au‐1) with high anisotropy (AR = 3.30) and, hence, our system exhibited better light‐harvesting properties compared to other literature‐known systems containing gold nanospheres. Moreover, the AuNRs covered red‐NIR spectral range inaccessible to Azo component (**Figure** **5**a), enabling efficient triggering of plasmon‐related effects. The NIR‐mediated catalysis is particularly beneficial for bio‐related research and was previously used, e.g., for photothermal cyclization^[^ ^78^ ^]^ or depropargylation^[^ ^79^ ^]^ reactions inside living cells using plasmonic nanoreactors. The advantages of our formulation guarantee that changes in the Azo *Z‐E* isomerization kinetics are not the result of direct triggering of Azo isomerization. To support our claims, we used a custom experimental setup with real‐time UV–vis–NIR spectra acquisition and sample irradiation cell integrated with LED light source and heating stage, to assure constant base temperature of 25 °C (Figure 5a, top). We selected 650–1100 nm spectral range (Figure S14, Supporting Information) corresponding to the position of the l‐LSPR band of Au‐1 plasmonic core (Figure 5a, bottom). The content of AuNRs in the hybrid formulation can be tuned according to the needs, however, to benefit from plasmon‐derived phenomena we increased the loading of CNFs with AuNRs to 19.6 wt.% (Figure S21, Supporting Information). Finally, we carried out two types of experiments to investigate the influence of AuNRs upon red‐NIR irradiation on 1) AzoGly in a form of free molecules in EtOH or 2) CNFs‐bound photochrome in the hybrid materials in water.

First, we investigated plasmon‐assisted *Z‐E* isomerization of free Azo molecules as a prelude to understanding this process in the hybrid material. By examining the reaction in the native Azo solvent (EtOH) we increased the temporal resolution of the measurement. In this experimental scenario, bare CNFs or Au‐1‐CNFs pre‐composite were added directly to the *Z*‐form of AzoGly, i.e., after UV irradiation. Temporal separation of Au addition from the UV exposure excludes possible participation of intraband excitations. The chosen irradiation conditions (650–1100 nm, 64.9 mW cm^−2^) and bare CNFs have a negligible effect on pristine Azo isomerization kinetics (Figure 5b; Figure S22, Supporting Information). Observed differences in *k_Z‐E_* values (dark vs light conditions as well as pristine Azo vs bare CNFs addition) are characterized by typical relative errors^[^ ^80^ ^]^ (see Section 5.1, Supporting Information). This confirms catalytically inert nature of CNFs scaffold. A significant catalytic boost was clearly observed once Au‐1‐CNFs pre‐composite was added to the PSS*_Z_* of AzoGly (Figure 5b). The rate constant value in the dark, *k_Z‐E,Au_^dark^* = (7.09 ± 0.04) × 10^−4^ s^−1^, was one order of magnitude higher compared to samples without gold. Furthermore, in the presence of Au and upon irradiation with red‐NIR, two orders of magnitude increase was observed, *k_Z‐E,Au_^light^* = (3.15 ± 0.03) × 10^−3^ s^−1^. Notably, the light‐assisted process exhibited first‐order kinetics, as confirmed by the monoexponential fitting of the data, which indicates that the catalytic boost derived from AuNRs is experienced uniformly by the whole population of Azox molecules.

We propose looking at this data through the lens of a photochrome. Hence, we used the parameters of the Arrhenius plot for a free AzoGly ($lnk\;=\;a\cdot \frac{1}{T}+b$, where *a* = −9224.0 [K], *b* = 19.2 [‐], according to Figure S19, Supporting Information) to recalculate the determined rate constant values in the presence of AuNRs (*k_Z‐E,Au_^dark^* and *k_Z‐E,Au_^light^*, Figure 5c) into temperatures in which equivalent Azo *Z‐E* isomerization in the dark and without Au would occur at the same rate. Hence, we defined the so‐called “virtual temperatures” of the process. In the dark and in the presence of AuNRs, the sole catalytic effect of electron transfer can be expressed by a virtual temperature of 76 °C (Figure 5c). Upon irradiation, when the thermoplasmonic effect is triggered, the process remains pseudo‐first order, and the rate constant, *k_Z‐E,Au_^light^*, translates into a virtual temperature of 97 °C (Figure 5c). We propose that these values constitute a quantitative measure of the catalytic effect of AuNRs and the difference between them (21 °C) reflects the contribution of the thermoplasmonic effect. The temperature rise by 21 °C can be explained by collective heating of AuNRs.^[^ ^81^ ^]^ Although the separation between nanoparticles in the composite might be too large for the optical coupling, it is still sufficiently small for diffusion‐driven collective heating, which uniformly affects photochromic component in the hybrid material. Certainly, more efficient electron transfer upon irradiation (due to interband excitations) cannot be entirely ruled out, however, we assume the contribution of photothermal effect to be more substantial. To confirm the mechanism of the plasmon‐assisted Azo *Z‐E* isomerization in the hybrid material, we plotted values of the reaction rate constant as a function of the power density of the incident irradiation. Under the applied continuous wave (CW) irradiation and moderate irradiation powers, the exponential fit of the data is in accordance with the Arrhenius law (Figure S23, Supporting Information), which indicates toward the dominant role of the thermoplasmonic effect.^[^ ^82^ ^]^ With these intriguing results, we foresee the use of azobenzenes as molecular thermometers capable of determining temperature changes close to the nanoscopic sources of heat, especially having in mind a vast library of available Azo compounds, which differ in solubility, grafting potential, and thermal stability. Hence, Azo derivatives can be incorporated in different types of materials and structures, asserting proper resolution and sensitivity of the measurement for specific systems and applications. The analysis of advantages and disadvantages of the proposed idea is presented in Table S3 (Supporting Information).

The second experimental scenario concerns plasmon‐assisted isomerization of the photochromic component in the AzoGly‐Au‐1‐CNFs hybrid material. We observed that the increase of the amount of Au results in a lower experiment resolution (Figure S21, Supporting Information) due to significant overlap of the π→π^*^ band of a photochrome with the increased scattering of Au‐1‐CNFs framework. Moreover, increased loading contributes to faster composite sedimentation and randomizes its macroscopic behavior. To minimize the possibility of systematic errors, account for random deviations stemming form high Au‐loading, and facilitate the depiction of material's susceptibility toward light, we designed an OFF–ON experimental approach for hybrid Azo‐Au systems. Composites were subjected to the consecutive intervals of dark and light conditions during individual measurement. Conditions were identical through the whole experiment and the only difference was either presence or lack of irradiation and triggered plasmon‐related effects. Thus, we propose an experimental design based on real‐time analytics aided by advanced statistical modelling.

After reaching PSS*_Z_* in the hybrid material, AzoGly back‐isomerization was monitored under the same‐length dark (OFF) and light (ON, red‐NIR irradiation, 650–1100 nm, 145.8 mW cm^−2^) intervals. The collected spectra were accordingly processed (see Materials and methods in the Supporting Information) to conform with the assumptions of the applied statistical modelling approach – interrupted time series^[^ ^83^ ^]^ (Figure 5d; Section 5.2, Supporting Information). We consider observations before and after an intervention, which is the introduction of light. Intervention occurs a few times during each experiment (irradiation source was switched on and off multiple times). Dark and subsequent light intervals are paired up (in this exact order) and each pair is considered separately for a given sample. Our central hypothesis is that in the presence of Au, due to the plasmon‐assisted Azo isomerization triggered upon irradiation, there are statistically significant differences within subsets (pairs) upon intervention (Figure 5d). Once thermoplasmonic effect is triggered and the collective heating of AuNRs causes temperature rise in the vicinity of the nanostructures, AzoGly as a T‐type photochrome should undergo *Z‐E* isomerization faster. Hence, the slope of the linearized data should increase in absolute value during light period (Figure 5d), which can be qualitatively assessed through naked eye inspection (Figure 5e). However, a non‐trivial composition of our materials requires appropriate statistical tools to extract quantitative data. Auto‐correlation of the observations violates the assumptions of classical linear models and to account for the temporal structure of the data we analyzed it with an Autoregressive Integrated Moving Average (ARIMA)‐based approach.^[^ ^58^ ^]^ ARIMA models represent a single variable of interest Y_t_ as a function of its previous values (e.g., Y_t‐1_), random error (ϵ_t_), and previous values of the random error (e.g., ϵ_t‐1_), seasonal effect, differencing, and optional covariates (Figure 5d,e; Section 5.2, Supporting Information).

We investigated four samples in total (Table S4, Supporting Information) – with AuNRs (1 and 2) and control samples without Au (3 and 4) – and maintained the same conditions for each light interval (650–1100 nm, 145.8 mW cm^−2^) changing only its duration – either 5.0 min (for 1 and 3) or 2.5 min (for 2 and 4). For each sample at least four OFF–ON pairs were modelled, corresponding to the time frame of the biggest extinction change. By comparing sample 1 (Au) and 3 (no Au) presented in Figure 5e (bottom and top, respectively), we recognize that intervention causes slope change only for sample containing AuNRs. This manifests as a characteristic breaking of the linear trend of the data at the times corresponding to the intervention (so‐called Ramp effect) and is in contrast to the overall smooth linear trend for the control sample without gold (Figure 5e, see also Figure S24, Supporting Information for samples 2 and 4). Based on the ARIMA modelling (for selected *p*, *d*, and *q* parameters, Table S5, Supporting Information), we estimated values of the Ramp effect upon intervention (Table S6, Supporting Information). Negative values imply that the extinction at the maximum of the π→π^*^ band is changing faster under irradiation. This means that AzoGly isomerization rate increases during light intervals comparing to dark conditions. Visual assessment and point estimates of the Ramp effect are, however, not sufficient for making statistical statements about the significance of the observed slope differences. After taking into account the uncertainty of estimation and correcting for multiple comparisons, we calculated p‐values for tests of significance of the Ramp effect (Table S7, Supporting Information). At 5% significance level, we treat effects with p‐values smaller than 0.05 as significantly different from 0. Testing results (Figure 5f) confirm that the statistical significance of the Ramp effect (slope change upon irradiation) is observed only for sample containing gold. There is no statistical significance of the Ramp effect values for sample without gold. Conclusions are identical for samples 2 and 4 (Figure S24, Supporting Information). Testing results prove that introduction of AuNRs enables indirect photocontrol over AzoGly isomerization kinetics in the hybrid material in an ON–OFF manner.

Interestingly, the statistical modelling can be also used to determine which plasmon‐related effect plays significant role in the presented indirect photocontrol of Azo isomerization. Since there are multiple light and dark periods, the intervention can be defined in two ways, as switching light on or switching it off. Thus, we defined new pairs, ON–OFF, starting from the first light period and pairing it up with the second dark interval. By modelling the Ramp effect for new pairs we determined how switching irradiation off affects the reaction kinetics, as manifested in the change of the slope of the data. Results presented in Tables S8–S10 and in Figures S25 and S26 (Supporting Information) indicate that switching irradiation off also results in the data slope change only in the presence of Au. However, the “OFF” intervention is more subtle once the irradiation intervals are shorter. For sample 2, with shorter light periods, p‐values are larger than the assumed p = 0.05 level. Because the Ramp effect detects an immediate change upon intervention, we conclude that for switching irradiation off, slope change is not as instantaneous as it is for switching irradiation on. Consideration of the time‐scale of the photoinduced kinetic changes is an important aspect enabling determination of the mechanism of plasmon‐assisted process.^[^ ^82^ ^]^ For the photochemical mechanism, due to the short lifetime of hot carriers (order of few tens of fs for Au), kinetic changes would be immediate for both types of intervention. In case of the thermoplasmonic effect, however, heat dissipation characterized by a much longer timeframe needs to occur, especially for such a complex material dispersed in water. Because we observed less pronounced slope change upon switching irradiation off for shorter intervals, we can assume the dominant contribution of the thermoplasmonic effect during plasmon‐assisted Azo isomerization. Therefore, we present for the first time, the possibility of the indirect kinetic photocontrol of Azo *Z*‐*E* isomerization in an ON–OFF manner via thermoplasmonic effect.

## 3 Conclusion

We demonstrated a method to efficiently incorporate plasmonic and photochromic units within one processable and robust material. By employing intermolecular interactions and utilizing cellulose nanofibers (CNFs) as a functional scaffold, we preserved the photoswitchability of the chosen Azo derivative (AzoGly) and maintained stable plasmonic properties of AuNRs. At the same time, we kept beneficial spectral separation of the optical features of components and presented catalytic influence of AuNRs on Azo isomerization in the dark, upon UV and blue light irradiation. Our study addressed several challenges set in the field of plasmonic‐photochromic hybrids by previous research groups. Namely, we showed, for the first time, an entirely water‐functional system that does not rely on covalent linkage between components, employs large, anisotropic nanoparticles, and operates without aggregation. We also provided experimental proofs for the literature hypothesis of the electron transfer‐based catalytic influence of AuNPs on Azo *Z‐E* isomerization in the dark. Moreover, by applying Au nanostructures with great light‐harvesting properties, we proposed light‐driven, plasmon‐assisted *Z*‐*E* isomerization of Azo. As revealed by statistical modelling, the thermoplasmonic effect, triggered upon red‐NIR irradiation in the presence of Au, grants an ON–OFF control over two modes of the plasmon‐derived catalytic boost of Azo *Z‐E* isomerization kinetics in the hybrid material. Our study shows perspectives for the ARIMA‐based statistical modelling approach in the analysis of real‐time spectroscopy data for experimental scenarios considering spectral changes occurring upon external intervention. We also propose the use of Azo derivatives as molecular thermometers to quantify extent of the thermoplasmonic effect. This work opens up perspectives for further development of multifunctional hybrid materials relying on plasmonic nanostructures and photochromes and methods enabling temperature assessment at the nanoscale. Our material design constitutes a convenient starting point for further advanced azobenzene‐related research and 3D printing of macroscopic light‐responsive structures with dual functionalities.

## Conflict of Interest

The authors declare no conflict of interest.

## Author Contributions

N.T.S. performed initial and final project conceptualization (all aspects, including in particular: material formulation, plasmon‐assisted process, and azobenzenes as nanothermometers); in all aspects, namely, material formulation, components identification, photoswitching experiments, thermal relaxations, plasmon‐assisted process: methodology development and application, investigation (performing all experiments and all data collection), formal analysis of all acquired data, all data curation; project administration; visualization (all figures and tables); wrote the original and final draft, wrote, reviewed and edited the final manuscript. M.S. designed and implemented an R language application for the analysis of real‐time acquired UV–vis spectroscopy data (“plasmon‐assisted” part); performed formal analysis – ARIMA modelling and statistical analysis; wrote – statistical section; supporting visualization (statistical part); reviewed the final draft. M.D. performed investigation – synthesis and identification of AzoGly; formal analysis – supporting analysis of the hybrid materials' thermal relaxations; reviewed the final draft. M.G. performed supporting conceptualization (“electron transfer” and “plasmon‐assisted” parts); methodology development (ON–OFF experiments); supporting analysis of the data related to the plasmon‐assisted part of the project; resources – provision of instrumentation necessary for the plasmon‐assisted part of the project; reviewed the original and final draft; supervision. K.M. performed initial project conceptualization; supporting analysis of the hybrid materials thermal relaxations; resources – provision of instrumentation necessary for the spectroscopic measurements; supervision; reviewed the final draft.

## Supporting information

Supporting Information

## Data Availability

The data that support the findings of this study are available from the corresponding author upon reasonable request.
