# Supplementary material for: Thermoplasmonic Effect Enables Indirect ON–OFF Control over the Z‐E Isomerization of Azobenzene‐Based Photoswitch
Source: Small. 2024 Sep 3;20(47):2404755. doi: 10.1002/smll.202404755 (PMC11579967; doi:10.1002/smll.202404755)
Supplement: Supplementary file 1 — Supporting Information [file SMLL-20-2404755-s001.pdf]

NANO · MICRO
small

Supporting Information

for *Small*, DOI 10.1002/smll.202404755

Thermoplasmonic Effect Enables Indirect ON–OFF Control over the Z-E Isomerization of Azobenzene-Based Photoswitch

Nina Tarnowicz-Staniak, Mateusz Staniak, Marta Dudek, Marek Grzelczak* and Katarzyna Matczyszyn**

Supporting Information

Thermoplasmonic Effect Enables Indirect ON-OFF Control over the Z-E Isomerization of Azobenzene-Based Photoswitch

Nina Tarnowicz-Staniak, Mateusz Staniak, Marta Dudek, Marek Grzelczak*, Katarzyna Matczyszyn**

Table of Contents

Materials and methods………………………………………………………………………… **S2**

Section 1. Starting components…………………………………………………………………. **S7**

Section 2. AzoGly-Au-CNFs composite composition analysis - IR spectra……………………. **S11**

Section 3. Optimization of the AzoGly-Au-CNFs composite preparation protocol…………… **S15**

Section 4. Photochromic properties of the AzoGly-Au-CNFs composites……………………... **S18**

Section 5. Plasmon-assisted isomerization of AzoGly……………………………………….. **S24**

References………………………………………………………………………………… **S33**

Materials and methods

• Chemicals

All of the chemicals and solvents were commercially available and were used without further purification if not stated otherwise. Gold(III) chloride trihydrate ($HAuCl_4{\cdot}3H_2O$), cetyltrimethylammonium bromide (CTAB), sodium borohydride ($NaBH_4$), L-ascorbic acid (AA), hydrochloric acid (HCl), and silver nitrate ($AgNO_3$) were purchased from Sigma-Aldrich. Cellulose nanofibers were supplied by CelluForce (Canada). Deionized water (Millipore Milli-Q grade) with a resistivity of 18.2 MΩ·cm was used in all AuNPs-related experiments.

• Synthesis of Azo Derivative (AzoGly)

General procedure for preparation of the target azobenzene molecule (**AzoGly**) as well as ^{1}H, ^{13}C NMR spectra and HRMS are reported in the **Section 1.1.**

• Synthesis of Gold Nanorods (AuNRs)

Au-1 and **Au-2** gold nanorods (AuNRs) were prepared using a Ag-assisted seeded growth method in the presence of AA [1–4].

A batch of **Au-1** nanorods was prepared according to the following protocol: in the first step seeds were prepared by reducing $HAuCl_4$ (0.025 mL, 0.05 M) with ice-cold $NaBH_4$ (0.3 mL, 0.01 M) in an aqueous CTAB solution (4.7 mL, 0.1 M) under vigorous stirring. The mixture was subsequently left for 30 min under mild stirring. Further, to the growth solution containing CTAB (175 mL, 0.1 M), $HAuCl_4$ (1.75 mL, 0.05 M), AA (1.31 mL, 0.1 M), and $AgNO_3$ (1.75 mL, 0.005 M), a seed solution (1.75 mL) was added. The mixture was left undisturbed at room temperature for at least 2 h. The final concentration of metallic gold was 0.0005 M. UV-Vis extinction spectrum of the sample is presented

in **Figure S4** together with the representative TEM image. The average length of the Au-1 nanorods was 35.3 ± 4.2 nm and the average diameter was 10.7 ± 2.1 nm (AR = 3.3). Size histogram is presented in **Figure S5**.

A batch of **Au-2** nanorods was prepared according to the following protocol: in the first step seeds were prepared by reducing $HAuCl_4$ (0.25 mL, 0.010 M) with ice-cold $NaBH_4$ (0.6 mL, 0.010 M) in an aqueous CTAB solution (9.75 mL, 0.10 M) under vigorous stirring. The mixture was subsequently left for 10 min under mild stirring, and later the solution was kept unstirred at 27°C for 2 h. Further, to the growth solution of Au-2 containing CTAB (8 mL, 0.1 M), $HAuCl_4$ (0.5 mL, 0.010 M), AA (0.08 mL, 0.1 M), HCl (0.2 mL, 1.0 M) and $AgNO_3$ (0.030 mL, 0.010 M), a seed solution (2.0 mL) was added. Finally, the growth solution was left undisturbed for at least 16–20 h at 27°C. As-prepared nanostructures were purified on the next day via centrifugation at 16 000g for 35 min. The supernatant was subjected to the second centrifugation step and the second pellet was combined with the first one and redispersed in water. Final concentration of metallic gold was 0.0007 M. UV-Vis extinction spectrum of the sample is presented in **Figure S4** together with the representative TEM image. The average size of the Au-2 AuNRs was as follows: average length – 38.9 ± 3.3 nm, average diameter – 18.3 ± 1.3 nm (AR = 2.13). Size histogram is presented in **Figure S5**.

- **Preparation of Au-CNFs Pre-composites**

Pre-composite materials were prepared according to our previously established protocol.[5] Solutions of as-prepared AuNRs were washed twice with water (7000g, 15 min for **Au-1** and 16 000g, 30 min for **Au-2**) and finally redispersed in 1 mM CTAB solution. Subsequently, solutions of AuNRs were titrated with the aqueous solution containing 0.5 wt % of CNFs under vigorous stirring at room temperature, keeping a volume ratio of AuNRs/CNFs = 2:1. Samples were prepared in such a manner, that the resulting material contained the desired mass of gold in relation to mass of CNFs (~19.6 wt % in case of **Au-1** and ~5.4 wt % in case of **Au-2**). The as-obtained samples were left until the next day for material to sedimentate. Collected pellets were further centrifuged two times (540g, 5 min) and redispersed in water to remove the excess of CTAB. The resulting **Au-CNFs pre-composites** were subjected to visual inspection and UV–Vis–NIR characterization.

- **Preparation of AzoGly-Au-CNFs Composites**

Phase separated and purified **Au-CNFs pre-composites** were centrifuged (780g, 5 min) and redispersed in ethanol. The as-obtained clear dispersions were subsequently added dropwise to the solution of **AzoGly** in ethanol under vigorous stirring. Concentration of AzoGly was adjusted to ~3.1 mM for all samples (see more details in the **Section 3.1.1.**). Mixtures were stirred for 5 min and then left undisturbed for at least 17.5 h at room temperature. After this time hybrid photochromic-

plasmonic composites were separated from ethanol via centrifugation at 540-1400g for 10-15 min. The resulting supernatants were subjected to up to three follow-up separations at 7000g for 10-20 min. All of the combined pellets were transferred to water as a solvent of choice and were subsequently washed once (4850g, 20 min) to remove the unbound excess of AzoGly and any remaining ethanol. The resulting composites were subjected to visual inspection, UV–Vis–NIR characterization, and further in-depth investigation.

- **Preparation of Control AzoGly-CNFs Composites**

To prepare composites without AuNRs for the control measurements, 1 mM CTAB solution was titrated with the aqueous solution containing 0.5 wt% of CNFs under vigorous stirring at room temperature, keeping a volume ratio of CTAB/CNFs = 2:1. The obtained sample was left until the next day for the material to sedimentate. Pellet was further centrifuged two times (540g, 5 min) and redispersed in water to remove the excess of CTAB. Third centrifugation (780g, 5 min) was performed to redisperse material in ethanol. The as-obtained clear dispersion was subsequently added dropwise to the solution of AzoGly in ethanol under vigorous stirring. For control samples concentration of AzoGly was always adjusted to ~3.1 mM – the same as for samples containing AuNRs. Mixture was stirred for 5 min and then left undisturbed for at least 17.5 h at room temperature. After this time mixture was purified and washed via centrifugation according to the procedure established above for the **AzoGly-Au-CNFs** composites.

- **Characterization of the Composite Materials**

UV–Vis–NIR extinction spectra measurements were performed using either JASCO V-730 spectrophotometer equipped with Peltier UV-Vis cuvette holder or Cary UV-Vis-NIR 3500 spectrophotometer equipped with Multicell Peltier UV-Vis cuvette holder. Transmission electron microscopy (TEM) images were acquired using Hitachi H-800 and JEOL JEM-F200 microscopes. The ATR FT−IR spectra were collected using a Bruker Vertex 70v Fourier transform infrared spectrometer equipped with an air-cooled DTGS detector and diamond attenuated total reflection infrared cell in the middle-infrared (4000 – 400 cm^{-1}) and far-infrared (600 – 100 cm^{-1}) regions at room temperature. Instrument control and initial data processing were performed using OPUS Software (v. 7.0, Bruker Optics, Ettlingen Germany).

- **Photostability and Photoswitching Tests of AzoGly-CNFs and AzoGly-Au-CNFs Composites**

For the irradiation experiments the Hamamatsu L9588 LightningCure LC8 spot light source was used. *E*-*Z* and *Z*-*E* isomerizations occurring in the Azo-containing composite materials (with and without AuNRs) were induced with UV irradiation (365 nm, irradiation intensity 1.93 ± 0.06 mW cm^{-2}) and

visible light (436 nm, light intensity 5.1 ± 0.1 mW cm^{-2}), respectively. Accompanying UV-Vis absorption measurements were carried out until reaching respective photostationary states. The exact wavelengths were selected using bandpass filters (**Figure S14**). Irradiation intensities were measured with Thorlabs S425C Thermal Power Sensor and Thorlabs PM100D Compact Power and Energy Meter Controller.

- **Thermal Relaxations – Half-Life Determination**

To analyze thermal relaxation of the AzoGly *Z*-isomer and its return to the *E*-form, the absorbance changes of the $\pi \rightarrow \pi^*$ band were measured as a function of time at different temperatures. For free AzoGly molecules, 38 μM solution in EtOH was used, while in case of the investigated composite materials (either **AzoGly-CNFs** or **AzoGly-Au-2-CNFs**), their dispersions in H_2O were used. First, solutions were irradiated with UV (365 nm for about 3-5 min) to ensure the conversion of *E*-isomer. Subsequently, the extinction changes at single point (corresponding to the maximum of the AzoGly $\pi \rightarrow \pi^*$ band) were measured as a function of time, and the readings were taken every 10 seconds. Measurements were done for four consecutive temperatures from the following set: 20, 25, 30, 35, and 40°C (starting either from 20 or 25°C) until samples relaxed to the *E*-form of Azo.

- **Plasmon-assisted isomerization of AzoGly**

Plasmon-assisted isomerization of AzoGly was investigated via UV-Vis-NIR extinction spectra measurements performed using Maya2000 Pro High-Sensitivity Spectrometer (Ocean Insight) coupled via fibre optics to DH-mini UV-Vis-NIR Deuterium-Halogen Light Source with shutter and UV-Vis cuvette holder. Samples were irradiated during measurements using G2V Pico Small Area LED Solar Simulator. Custom irradiation programmes were applied and their parameters (spectral range and irradiation intensities) are specified in the experiments descriptions in the main text or Supporting Information accordingly. Temperature of the samples was adjusted to room temperature (25°C) using heating stage controlled by Linkam MC60 controller.

Au-CNFs material and free AzoGly in solution – continuous irradiation conditions. Ethanol solution of AzoGly (0.1 mM) was irradiated with UV (370 nm, 2.2 mW cm^{-2} for about 5 min) to ensure the conversion of AzoGly *E*-isomer. Subsequently, to the *Z*-rich AzoGly solution **Au-1-CNFs** material was added (2:1, v/v). Sample was subjected to relaxation either in the dark or upon red-NIR irradiation (650-1100 nm, 64.9 mW cm^{-2}). In both variants experimental data (extinction spectra) were collected every 1 second for 1 hour. Experiments were repeated for control samples without gold (bare CNFs).

Plasmonic-photochromic composite materials – OFF-ON interval experiments. Water dispersions of **AzoGly-Au-1-CNFs** composites were irradiated with UV (370 nm, 2.2 mW cm^{-2} for about 5 min) to ensure the conversion of AzoGly *E*-isomer. Subsequently, samples were subjected to interval dark and red-NIR (650-1100 nm, 145.8 mW cm^{-2}) conditions in an OFF-ON manner, to evaluate the influence of photocatalytic and photothermal effect of gold nanoparticles on the ongoing photochromic reaction. Experimental data (extinction spectra) regarding AzoGly relaxation in the changing conditions were collected every 1 second at least for fourteen consecutive intervals (seven pairs of the OFF-ON intervals). Experiments were repeated for control samples without gold (**AzoGly-CNFs** composites).

• Statistical Analysis of Interrupted Time Series Experiments (OFF-ON Interval Experiments)

Collected extinction spectra were transformed in the following way. Firstly, extinction values at wavelengths corresponding to the maximum of the $\pi \rightarrow \pi^*$ band of AzoGly were extracted as a function of time. Thus, for each sample, dataset were represented as a sequence (t, Ext_t), where t – time [s], Ext_t – extinction value [-] measured at that time. Secondly, each dataset was linearized according to the formula: $Y_t = ln\left(\frac{Ext_\infty - Ext_t}{Ext_\infty - Ext_0}\right)$, where Ext_∞ - extinction before UV irradiation, Ext_0 - extinction right after UV irradiation. To account for the temporal structure of the data, an Autoregressive Integrated Moving Average (ARIMA)-based approach[6] was applied for interrupted time series designs. Interrupted time series design describes experiments where for a given subject, observations are collected before and after an intervention. Thus, dataset is split into sub-series. In this case the intervention of interest was the introduction of light or turning the light off. Moreover, since the light is switched on and off multiple times for each sample, each pair of the following dark and light (or light and dark) segments for a given sample was considered separately. To estimate the parameters of ARIMA model including the Ramp effect, which corresponds to change in slope of the data after intervention, the auto.arima function of forecast package[7] available in the R statistical environment[8] was used. The auto.arima function estimates the unknown effects using maximum likelihood approach (ML) and, thus, significance testing stemmed from standard ML-based methods. The ARIMA model was fitted for each pair of the following dark and light (or light and dark) time segments for each sample. Then, for each model the significance of Ramp effect was tested, which enabled detection of the change in slope occurring after intervention. To account for multiple comparisons in each sample the Benjamini-Hochberg correction[9] was applied.

Section 1. Starting components

1.1. AzoGly Ligand

1.1.1. Synthesis

Synthetic and Analytical General Methods

Solvents and reagents were purchased from commercial suppliers and were used as supplied. Column chromatography was performed using silica gel (Acros 60, 40–60 mesh). NMR-spectra were recorded on a Bruker AvanceTM 600 MHz spectrometer or on a JEOL 400 MHz spectrometer at 25°C using residual protonated solvent signals as internal standards for ^{1}H- and ^{13}C-spectra. High resolution mass spectra (HRMS) were conducted with a WATERS LCT Premier XE mass spectrometer (ESI).

Composition of the photostationary states (PSSs)

The photoinduced isomerization reactions of AzoGly were performed by using a UV Spot Light Source (Hamamatsu Photonics K.K., model: L9588-04) equipped with filters operating at: 365 nm and 436 nm. The composition of the photostationary states (PSS_Z and PSS_E) was determined by ^{1}H NMR spectroscopy by irradiating the sample (8.8 mM in CD_3OD solution at 25 °C) each time for 10 minutes, with different excitation sources: 365 nm and 436 nm, prior recording the NMR spectra. The composition of the PSS was calculated from the intensity ratios of the integrals of the corresponding peaks.

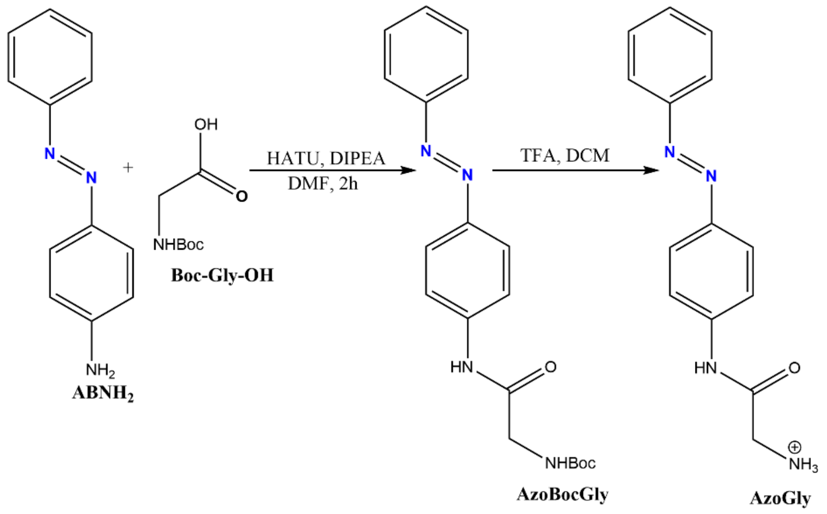

Scheme S1. *Synthetic route of AzoGly.*

AzoBocGly

To a solution of Boc-Gly-OH (0.32 g, 1.83 mmol, 1.2 equiv.) in DMF (10 mL), DIPEA (0.17 g, 1.3 mmol, 2.4 equiv.), HATU (0.69 g, 1.83 mmol, 1.2 equiv.) were added, soon afterward addition of 4-aminoazobenzene (0.30 g, 1.52 mmol, 1 eq.) then the mixture was stirred for 2 h at rt. Next, the water was added to the solution and the formed precipitate was filtered off, washed with water, and

dried. The crude residue was purified by column chromatography on SiO_2 gradient from DCM to 1% MeOH in DCM to give AzoBocGly as an orange powder (0.4 g, 74%).

^{1}H NMR (601 MHz, Methanol-*d*$_4$) δ: 7.93 – 7.86 (m, 4H), 7.80 – 7.75 (m, 2H), 7.55 – 7.45 (m, 3H), 3.90 (s, 2H), 1.48 (s, 9H).

HRMS m/z (ESI): $C_{19}H_{22}N_4O_3$ $[M+H]^+$, calculated: 355.1770, found: 355.1541.

AzoGly

To a solution of AzoBocGly (0.1 g, 0.18 mmol, 1 eq.) in DCM (10 mL) trifluoroacetic acid (0.41 g, 3.6 mmol, 20 eq.) was added. The mixture was stirred overnight, and then the solvent was removed under reduced pressure to give AzoGly as TFA salt form as an orange solid (quantitative).

^{1}H NMR (601 MHz, Methanol-*d*$_4$) δ: 7.93 – 7.87 (m, 4H), 7.81 – 7.79 (m, 2H), 7.55 – 7.47 (m, 3H), 3.47 (s, 2H).

^{13}C NMR (101 MHz, Methanol-*d*$_4$) δ: 164.4, 152.7, 149.0, 140.7, 130.8, 128.9, 123.5, 122.4, 119.6, 40.5.

HRMS m/z (ESI): $C_{14}H_{14}N_4O$ $[M+H]^+$, calculated: 255.1346, found: 255.1345.

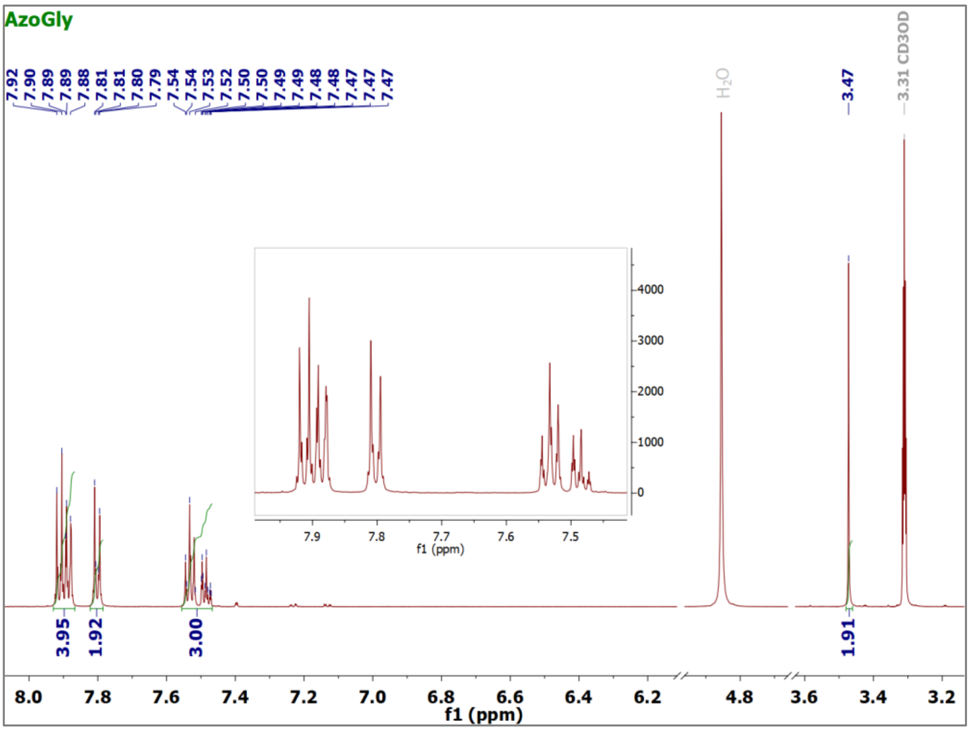

Figure S1. *^{1}H-NMR spectra of AzoGly in CD_3OD.*

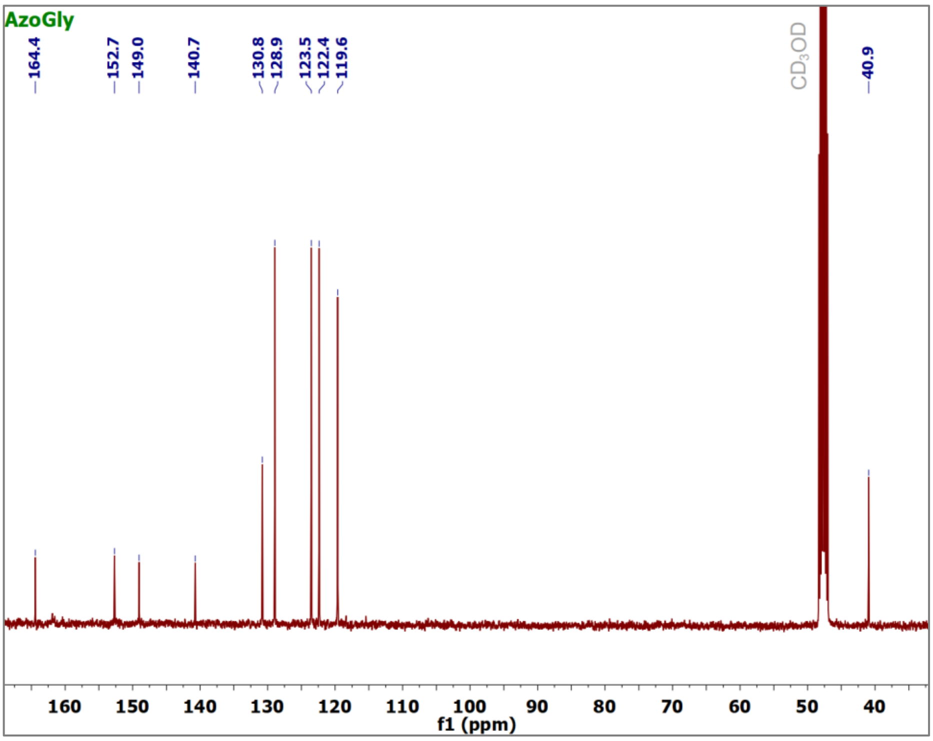

Figure S2. *^{13}C-NMR spectra of AzoGly in CD_3OD.*

1.1.2. Structure and properties

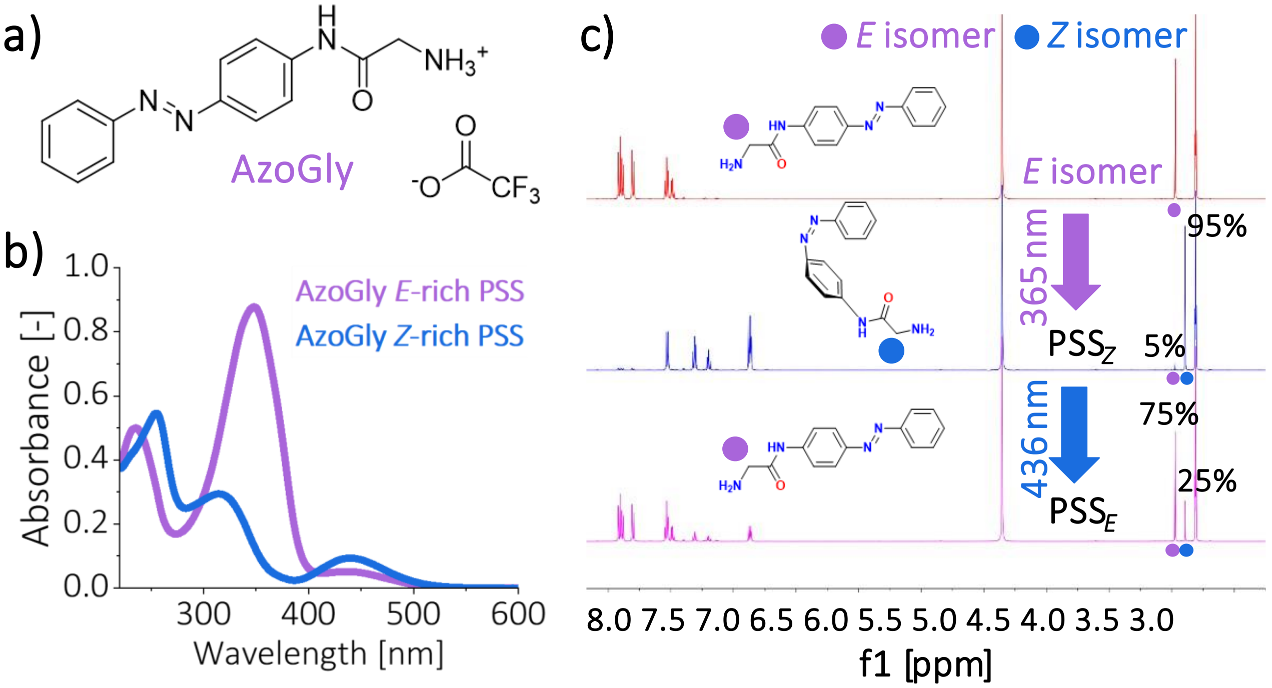

Figure S3. a) *Chemical structure of the target compound – AzoGly (in all experiments molecule was used in a form of salt with trifluoroacetic acid) and its* **b)** *UV-Vis absorption spectra in ethanol (both photostationary states: E-rich PSS_E and Z-rich PSS_Z).* **c)** *Quantification of the composition of the PSS_E and PSS_Z of AzoGly by ^{1}H NMR, based on the intensity ratios of the integrals coming from the E- and Z-isomer.*

1.2. Properties of Gold Nanorods (AuNRs)

In the course of the research project two different batches of AuNRs were synthesized, namely **Au-1** and **Au-2**. Au-2 nanoparticles were used in the experiments focused on the design and characterization of the original hybrid plasmonic-photochromic formulation. Au-1 nanorods were used in the ultimate part of the project regarding the indirect, plasmon-induced, ON-OFF control over AzoGly isomerization.

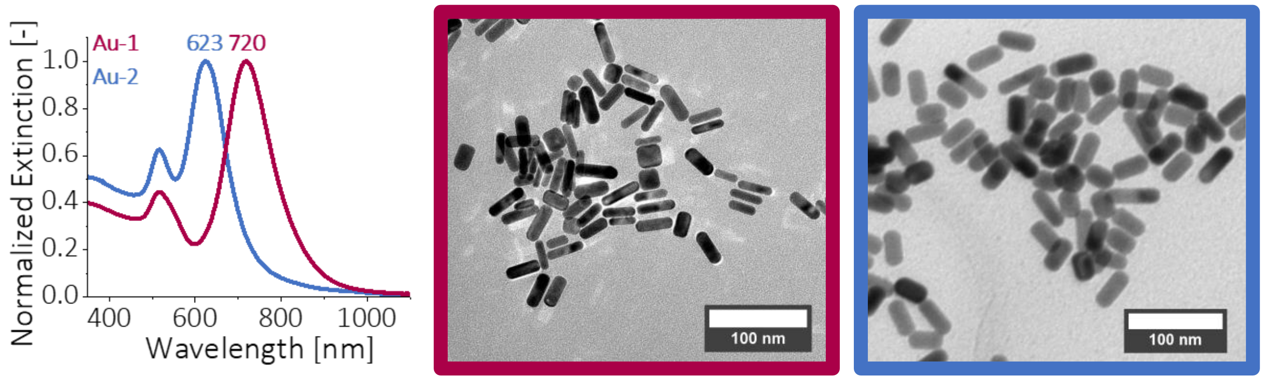

Figure S4. (left) *Normalized UV-Vis extinction spectra of the as-synthesized* ***Au-1*** *and* ***Au-2*** *nanorods with indicated positions of their longitudinal Localized Surface Plasmon Resonance (l-LSPR) bands.* **(middle, right)** *Appropriate TEM images for each sample. Colour codes of the plot lines, fonts, and frames are matching.*

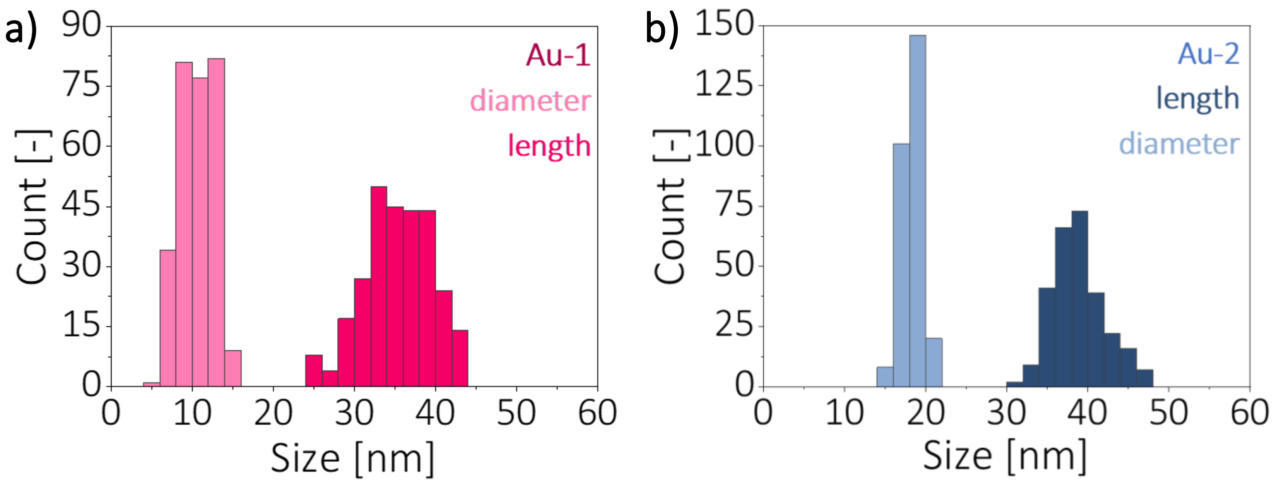

Figure S5. *Histograms presenting size distribution of the as-synthesized:* **a)** ***Au-1*** *nanorods, average length: 35.3 ± 4.2 nm; average diameter: 10.7 ± 2.1 nm, number of nanoparticles measured N = 277; aspect ratio AR = 3.30;* **b)** ***Au-2*** *nanorods, average length: 38.9 ± 3.3 nm, average diameter: 18.3 ± 1.3 nm, number of nanoparticles measured N = 275; aspect ratio AR = 2.13.*

1.3. Solubility of AzoGly

To verify the solubility of AzoGly, compound was dissolved in water and in ethanol to obtain ~0.7 mM solutions (the lowest concentration of AzoGly used later during the impregnation step) and was subsequently dilluted to ~30 μM, to obtain Absorbance < 1.0. Compound dissolved in ethanol easily but was not soluble in pure water and the aggregation of AzoGly was observed.

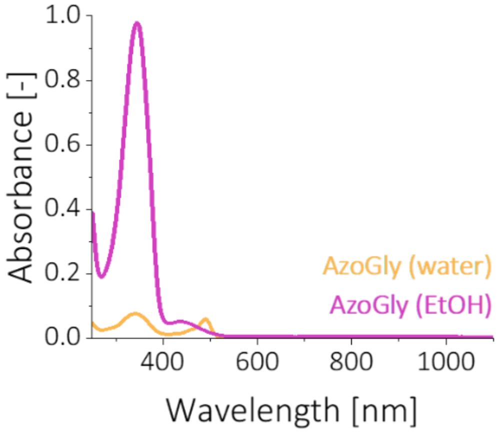

Figure S6. *Comparison of the UV-Vis-NIR absorption spectra of AzoGly solutions in water and ethanol. 30 μM AzoGly solution in ethanol was prepared easily and exhibited typical optical characteristics of the azobenzene-type photochromic compound.*[10] *It was not possible to prepare AzoGly solution in water without aggregation of the compound, which was reflected in the distorted profile of the spectrum even at low concentration.*

Section 2. AzoGly-Au-CNFs composite composition analysis - IR spectra

To facilitate IR spectra analysis, chemical structures of the initial components and pre-composite are presented in **Figure S7** below. Chemical structure of the final formulation is presented in **Figure 3b** in the main text. Chemical groups identified on the IR spectra (**Figure 3c**) are summarized in **Table S1** and highlighted in **Figure S7** and **Figure 3b**. Colour codes between figures are matching.

2.1. Important context

(1) AzoGly was used in a form of salt with trifluoroacetic acid (TFA), hence the counterion – trifluoroacetate – is also depicted in **Figure S7**. However, its $-COO^{-}$ group is not highlighted to not induce any confusion with the $-COO^{-}$ groups of CNFs. The counterion is not interacting with the composite material (rich on its own in $-COO^{-}$ groups), remains in the solution, and hence is removed during material purification procedure.

(2) As it is described in the main text, during pre-composite preparation CTAB is present both on the Au surface and as micelles in the solution. Both CTAB forms can interact with CNFs, and

both forms are presented in **Figure S7** – one of the molecules is ‘attached’ to the depiction of AuNR, the other is not. According to our hypothesis, CTAB serves as a molecular glue and may partially remain in the material after transfer to EtOH and also after impregnation with AzoGly. Surfactant serves as an additional stabilizer of the components and does not compromise the impregnation process.

2.2. Spectra analysis

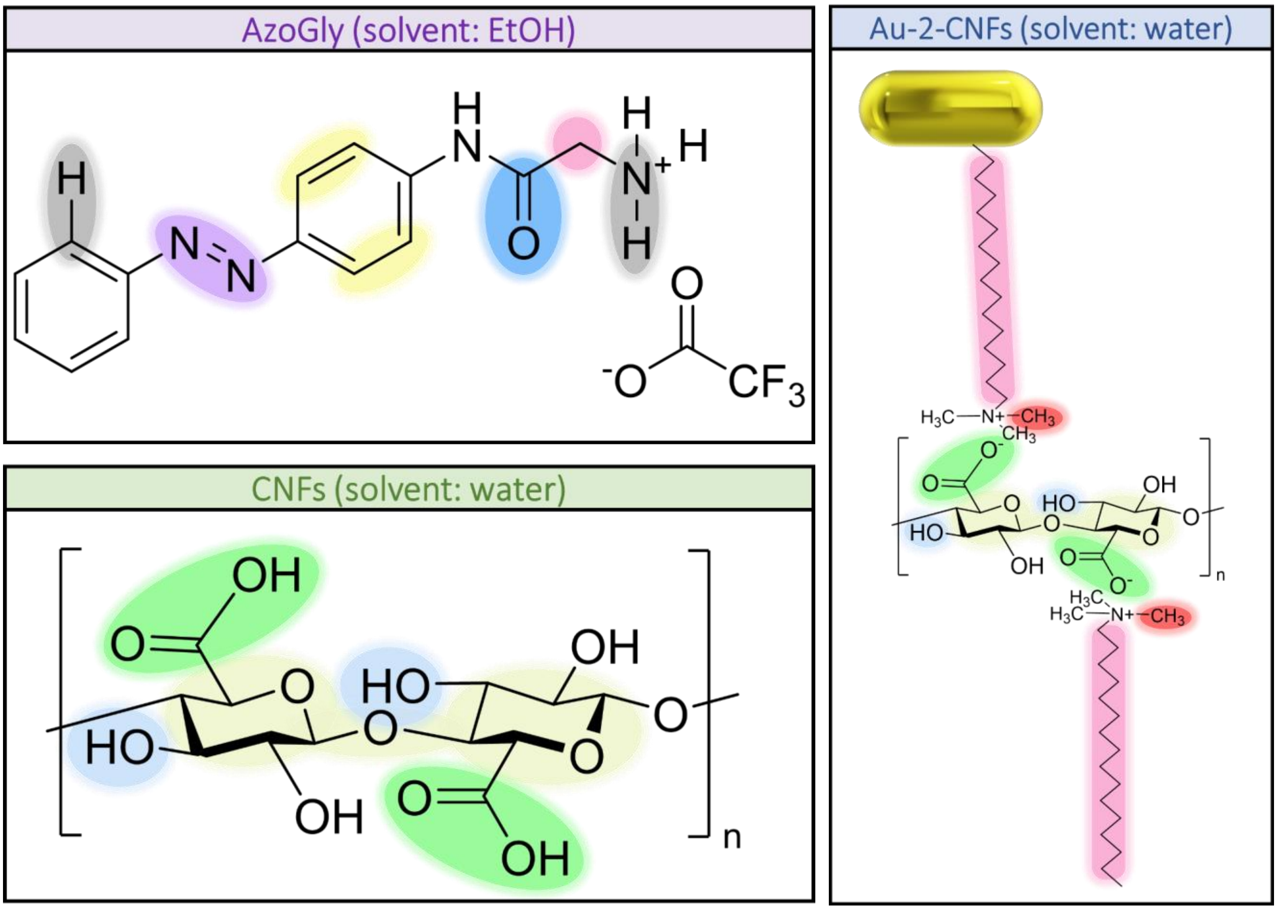

Figure S7. *Chemical structures of the initial components (AzoGly in ethanol and CNFs in water) and proposed structure of the Au-CNFs pre-composite in water (two possible forms of CTAB – free and bilayer on AuNRs – are indicated).*

Table S1. *Summary and interpretation of the IR signals identified on the spectra presented in* **Figure 3c** *(main text). Abbreviations mean as follows: ν – stretching vibration, $ν_s$ – symmetrical vibration, $ν_{as}$ – asymmetrical vibration, γ – out of plane deformation vibration, δ – in-plane deformation vibration.*

**Assignment**	**Band position [cm^{-1}]**			
	**AzoGly (@EtOH)**	**CNFs (@water)**	**Au-2-CNFs (@water)**	**AzoGly-Au-2-CNFs (@water)**
	**Cellulose identification**			
$O-H$ (ν)	3000-3500 (center: 3300, weak, round)	3000-3500 (center: 3338)	3000-3500 (center: 3344)	3000-3500 (center: 3342)
$-COO^{-}$ ($ν_{as}$)	1699 (trifluoroacetate present)	1641	1653 (weak)	part of 1653 (broad)
$-COO^{-}$ ($ν_s$)	1409 (trifluoroacetate present)	1427	1429	1419
$C-C$ ring (ν)	-	1159	1161	1161
$C-O-C$ pyranose ring ($ν_s$)	-	850-1200 (center: 1032)	850-1200 (center: 1059)	850-1200 (center: 1059)
$C-O-C$ β-glycosidic linkages	-	899	910 (weak)	905 (weak)
$O-H$ (γ)	-	663	667 (weak)	667 (weak)
$O-H$ bending of the absorbed water	-	1641	1653 (weak)	part of 1653 (broad)
	**CTAB identification**			
$C-H$ ($ν_s$)	2852	-	2858 (weak)	2854 (weak)
$C-H$ ($ν_{as}$)	-	2897	2922	2918
$-CH_3$ (ν) of ammonium salt	-	-	1456 (weak, part of 1429)	1456 (weak, part of 1419)
$C-N$ (ν, weak, shoulder)	-	-	906 (very weak)	906 (very weak)
	**AzoGly identification**			
$N=N$ (ν)	1545	-	-	1541 (part of 1653)
$C=C$ (ν) in plane	1599	-	-	1558 (part of 1653)

$C = O$ (ν)	1666	-	-	1653
$-N - H$ (ν) amine salt (strong) and $=C - H$ (ν, medium)	2924	-	2922	2918
$= C - H$ (γ)	768	-	-	758

2.2.1. CTAB as potential molecular 'glue' – a closer look

CTAB is an important component during synthesis of AuNRs and remains in the sample thereafter in a form of a stabilizing bilayer on the surface of Au and in a form of micelles in the solution. Its stabilizing shell grants AuNRs their positive charge. In our protocol, CTAB mediates the formation of **Au-CNFs** pre-composite, as well as serves as a molecular 'glue' partaking in providing material's structural integrity after transfer to EtOH. Its presence within material's structure after every step of the preparation protocol can be confirmed by the IR spectra, based on the $-CH_3$ stretching band of the ammonium salt (polar head of the CTAB molecule), typically appearing around 1450 cm^{-1}.

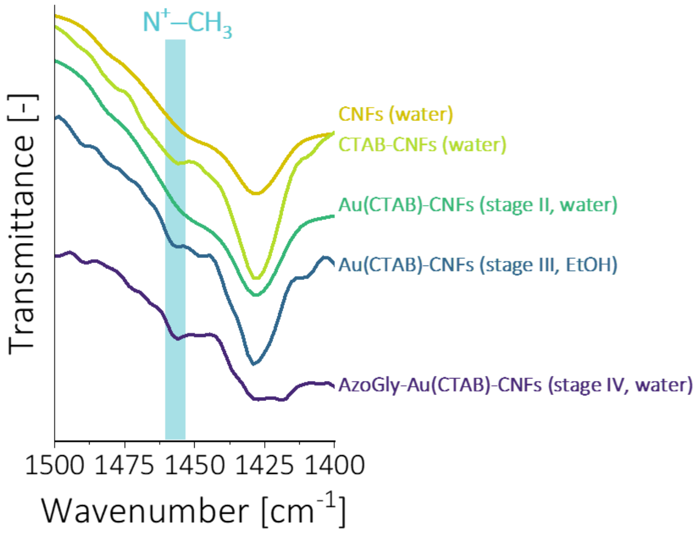

Figure S8. *Comparison of the IR spectra of the pre-composite before and after solvent transfer – Au(CTAB)-CNFs in water and Au(CTAB)-CNFs in EtOH, respectively. Spectra of bare CNFs in water, CNFs with the adsorbed CTAB, and final photochrome-containing material, AzoGly-Au-CNFs (all in water). Presence of the* $-CH_3$ *stretching band of the ammonium salt ~1456 cm*$^{-1}$ *confirms presence of CTAB throughout all protocol steps.*

Section 3. Optimization of the AzoGly-Au-CNFs Composite Preparation Protocol

3.1. Introductory information

Preparation of the AuNRs-CNFs composite has been optimized in our previous work.[5] In this section we present a detailed look into two control experiments: cellulose impregnation with dye without AuNRs and with thiolated Azo ligands.

3.1.1. Calculations – general remarks

The amounts of AzoGly used during the impregnation step were in the 2.7-3.4 mM concentration range, to maximize the photochromic properties of the composite (see **Figure S9** further in the text). The amount of AzoGly used in the impregnation step can also be expressed as a number of Azo molecules per nm^2 of total Au surface (~190 molecules/nm^2 for **Au-1** and ~900 molecules/nm^2 for **Au-2** nanorods). Calculations for the established protocol relied on the following information derived from literature.

(a) The extinction of AuNPs at 400 nm – Ext(400) – is representative of the total concentration of atomic gold[11,12] – [Au^0] (mM). At 400 nm the extinction coefficient is almost not dependent on the size of a gold nanoparticle.[13] Comprehensive studies have been presented for spherical[12] as well as rod-like AuNPs,[14] and in 2022, a new updated formula has been published that enables calculation of [Au^0] based on the data obtained in a simple UV-Vis absorption spectra measurement.[15]

Equation S1. $[Au^0]\ (mM) = Ext(400) \times 0.44$

Since all of the AuNRs introduced in the preparation step of **AuNRs-CNFs** pre-composite are immobilized on the cellulose fibers,[5] based on this assumption, [Au^0] can be recalculated into the total number of gold atoms in the sample – N_{Au}.

(b) As confirmed by the TEM pictures, the rod-like shape of AuNPs was assumed. We also assumed the bulk crystal structure of gold, FCC, with a unit cell of a dimension a = 0.408 nm (V = 0.0679 nm^3), and the number of Au atoms per unit cell equal to 4.[16–18] Combining these data with the volume of a single nanostructure (calculated for sizes estimated from TEM images) and N_{Au}, enables the calculation of the total number of AuNRs in the solution – N_{AuNRs}.

(c) Based on the surface of a single nanoparticle – S_{AuNR} (calculated for sizes estimated from TEM images) – as well as the N_{AuNRs}, the total surface of gold in the sample – S_{Au} – can be calculated. This enables determination of the number of AzoGly molecules per total Au surface.

3.2. Material impregnation with AzoGly – a detailed look

To investigate the potential of CNFs to adsorb AzoGly molecules bare CNFs were precipitated from the solution using CTAB. Subsequently, such pre-composite without AuNRs was transferred to ethanol and impregnated with AzoGly, using solutions of the photochrome with concentrations ranging from 0.00 mM to 2.62 mM. The as-obtained **AzoGly-CNFs** materials were purified and transferred to water. We observed that the material's separation efficiency differs, depending on the concentration of AzoGly used for the impregnation. For lower concentrations material separation was more difficult and the efficiency of the combined purification and transfer to water was significantly lower. For higher concentrations material separation occurred easily. We conclude that higher AzoGly concentration during impregnation results in a bigger population of the photochromic molecules adsorbed on CNFs, and hence in higher hydrophobicity of the fibers. The more hydrophobic the fibers, the more efficient their separation. UV-Vis-NIR absorption spectra of the as-prepared **AzoGly-CNFs** are presented on the **Figure S9a**. Clear tendency can be observed: the higher the concentration of AzoGly the higher the intensity of the $\pi \rightarrow \pi^*$ band (**Figure S9b** – direct values). To better visualize tendencies we adjusted spectral profiles of **AzoGly-CNFs** at 600 nm (below 600 nm the contribution of CNFs scattering becomes significant). Each sample was adjusted separately by subtracting spectral profile of bare CNFs (adjusted at $Ext(600)$) from that of a particular sample (**Figure S9a**). Corrected values of the intensities at wavelength corresponding with the $\pi \rightarrow \pi^*$ band of AzoGly were plotted against the concentration of the photochrome and the increasing trend was maintained (**Figure S9b** – corrected values).

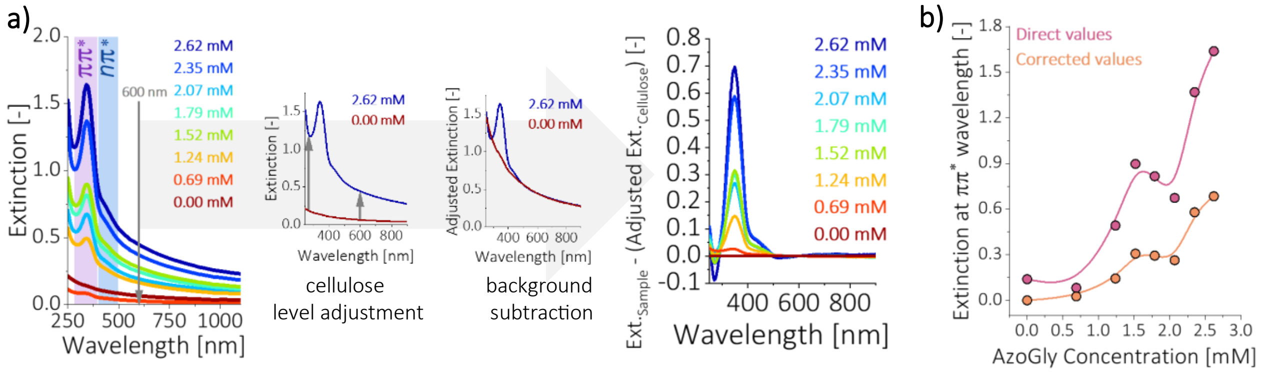

Figure S9. *Optical properties of the control samples without gold, AzoGly-CNFs.* **a)** *Schematic depiction of the correction approach and resulting spectra for all of the control samples.* **b)** *Dependency between extinction at wavelength corresponding with the maximum of the $\pi \rightarrow \pi^*$ band and AzoGly concentration during impregnation procedure.*

3.3. AzoGly vs. sulphur-containing ligands

Most of the Azo-Au hybrid preparation protocols rely on the chemical bonding of the two components by utilizing thiolated Azo molecules. To fully investigate how our preparation protocol compares

with typical Azo-Au hybrids preparation approaches, we decided to use sulphur-containing ligands (**Figure S10**) instead of AzoGly. The first ligand, **AzoSH**, has typical structure of the ligands used in other literature reports concerning Azo SAMs (self-assembled monolayers) on AuNPs.[19–21] The second ligand, **AzoSS**, was previously used for preparation of SAMs on planar gold surfaces.[22,23]

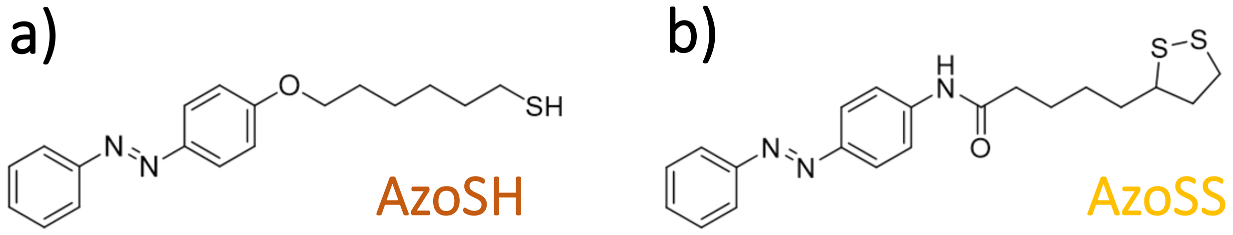

Figure S10. *Chemical structures of the sulphur-containing photochromic ligands* **a)** *AzoSH and* **b)** *AzoSS.*

Since in our approach the **Au-CNFs** pre-composite is transferred to EtOH, most of the Au surface is cleared from CTAB bilayer and thus accessible to potential Au-S interactions. Taking into account typical surface area occupied by a single Azo molecule,[24] we adjusted AzoSH and AzoSS concentrations to correspond to ~200-fold excess of ligand per binding site on **Au-2** core.

(1) Control experiment with AzoSH ligand

AzoSH is soluble in THF. Since THF and EtOH are well miscible, we carried out the impregnation by mixing AzoSH solution in THF with **Au-CNFs** pre-composite dispersed in EtOH. After the overnight impreganation, the resulting hybrid material was heavily aggregated as presented in **Figure S11a**. Material did not exhibit plasmonic or photochromic properties as presented in **Figure 3d** in the main text (due to the significant scattering of aggregates, the spectral profile of the sample was multiplied by 0.01). This indicates total aggregation of the material and, hence, the approach was deemed unsuccessful.

(2) Control experiment with AzoSS ligand

AzoSS was soluble in EtOH and hence, the impregnation process was performed exactly as for AzoGly. After the overnight impregnation process, material was clearly aggregated which, similarly to the control experiment with AzoSH, was reflected in the spectral profile of the sample (**Figure S11b**). Due to the highly scattering nature, the extinction profile of the sample was multiplied by 0.1. Material exhibited photochromic bands characteristic for AzoSS and even a small bump close to the position of the l-LSPR band of **Au-2** core. However, due to the aggregated form of the sample, material's plasmonic properties were almost non-existent. Since the resulting control composite did not exhibit dual functionalities and was aggregated in water, this approach was also deemed unsuccessful.

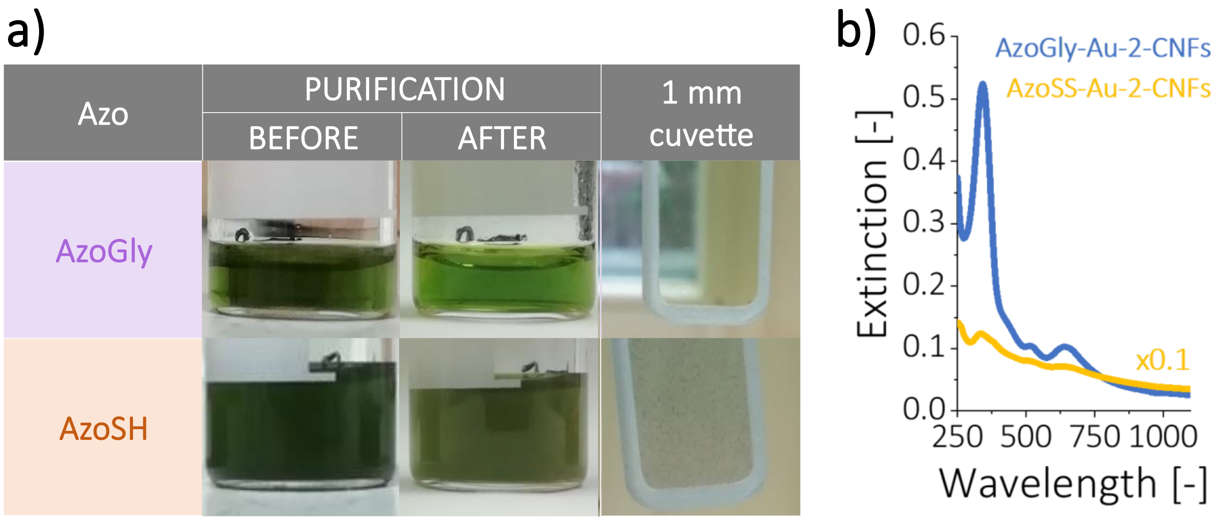

Figure S11. a) *Visual comparison of the prepared samples using either AzoGly or AzoSH, depicting material's aggregation after impregnation with AzoSH. Aggregates are clearly visible for the image in the '1 mm cuvette' column. Cuvette internal width is 10 mm.* **b)** *Sample impregnated with AzoGly (proposed approach) vs. AzoSS (control sample) – comparison of the spectral profiles. Due to the highly scattering nature of the control sample the extinction values were multiplied by 0.1.*

Comparison of the results obtained for both sulphur-containing ligands with our approach clearly shows advantages of the proposed material design.

Section 4. Photochromic properties of the AzoGly-Au-CNFs composites

4.1. Initial information regarding light-driven phenomena

Experiments focused on the light-driven processes occurring within the composite can be divided into two separate categories. First category is centred around **basic photochromism of the samples**, derived from the presence of AzoGly and will be discussed in **this section**. Second category of experiments is focused on the **potential plasmon-assisted isomerization of Azo** and will be discussed in **Section 5**.

4.1.1. Stability of the plasmonic properties

The optical properties of AuNRs incorporated in the final material are maintained.

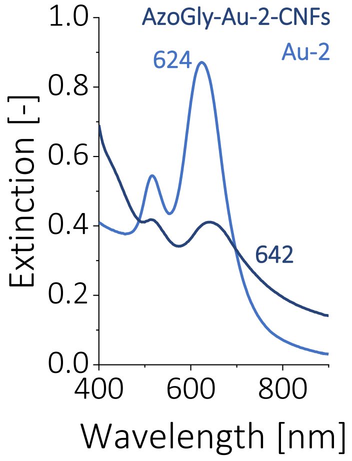

Figure S12. *Comparison of the l-LSPR band position of the hybrid sample vs. bare* ***Au-2*** *plasmonic core (18 nm redshift).*

4.1.2. Defining spectral regions of the samples

The influence of light of different wavelengths on the **AzoGly** isomerization was investigated. Due to close proximity of the t-LSPR band of AuNRs to the n→π^* band of **AzoGly**, emphasis has been put on the *Z-E* isomerization reaction. Hence, **AzoGly** solution in EtOH was irradiated with UV to induce *E-Z* isomerization and, subsequently, sample was monitored upon irradiation at 500-800 nm (>500 nm), 550-800 nm (>550 nm), and 600-800 nm (>600 nm). Results were compared with Azo isomerization in the dark and upon 436 nm irradiation. Irradiation was performed using Hamamatsu L9588 Lightningcure LC8 spot light source and spectral regions of irradiation were selected using colour glass filters. Typical photoswitching experiments were induced using UV (365 nm, irradiation intensity 1.93 ± 0.06 mW cm^{-2}) and visible light (436 nm, light intensity 5.1 ± 0.1 mW cm^{-2}). Irradiation intensity for the experiments performed in the spectral ranges >500 nm, >550 nm, and >600 nm was around 200-300 mW cm^{-2}.

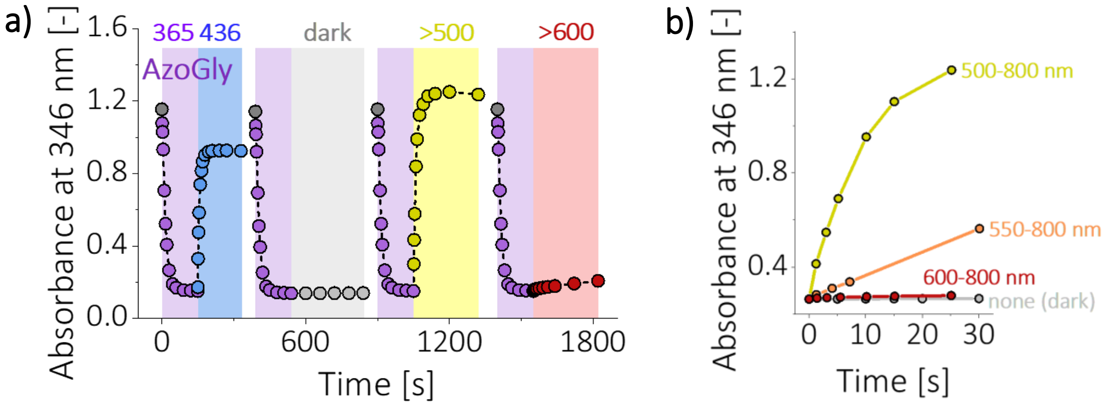

Figure S13. a) *Absorbance changes at the maximum of the $\pi \rightarrow \pi^*$ band of AzoGly (at 346 nm) as a function of time and irradiation conditions indicate that the Z-E isomerization of AzoGly can still be triggered by irradiation starting around 500 nm, however not by irradiation starting above*

600 nm. **b)** *To narrow down the best spectral range an additional relaxation upon 550-800 nm (>550 nm) irradiation was monitored, however in a very short time of 30 seconds a significant deviation from the dark conditions was observed. Hence, only the spectral range >600 nm was determined to not trigger the Z-E isomerization of AzoGly.*

For the irradiation carried out >600 nm (600-800 nm), the irradiation profile resembled dark (purely thermal) *Z-E* isomerization. We ascribed a small increasing tendency within the 5 min of the experiment (compared to the dark conditions) to **temperature change of the sample**. We measured temperature inside of the cuvette upon irradiation using PT1000 temperature sensor and observed temperature rise by 5°C. By adjusting the irradiation intensity, and by proper temperature control of the sample, 600-800 nm spectral range can be successfully used for the irradiation of the longitudinal plasmon bands of AuNRs without direct triggering of the AzoGly isomerization. **Hence, in the further presented plasmon-assisted experiments, irradiation spectral range starts above 600 nm.**

4.1.3. Irradiation conditions

Basic photochromism of the samples was investigated upon irradiation with Hamamatsu L9588 Lightningcure LC8 spot light source. Appropriate irradiation conditions were selected using optical filters (**Figure S14a**). Plasmon-assisted isomerization of Azo (discussed in the follow-up section of the SI) was carried out using custom selected programmes of G2V Pico Small Area LED Solar Simulator (**Figure S14b**). The advantage of the chosen LED light source lies in the fact that light beam does not affect the temperature of the samples during red-NIR irradiation.

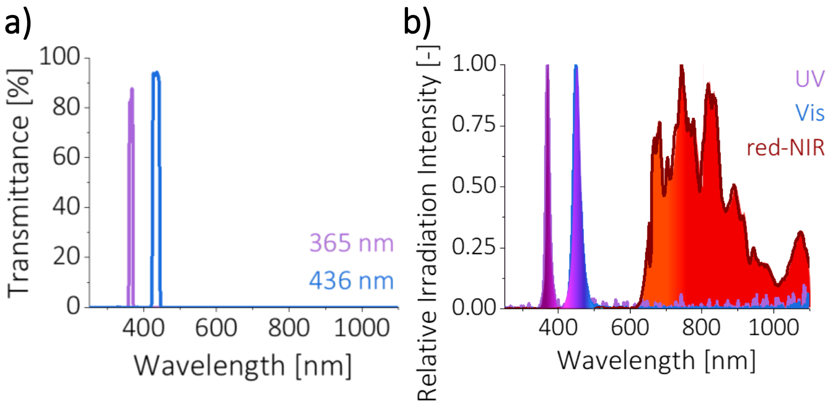

Figure S14. a) *Transmission spectra of the filters used for basic photoswitching and photostability experiments.* **b)** *Irradiation conditions during plasmon-assisted Azo isomerization experiment, depicted as spectral profiles of the light source (G2V Pico Small Area LED Solar Simulator).*

4.2. Photostability and photoswitching experiments

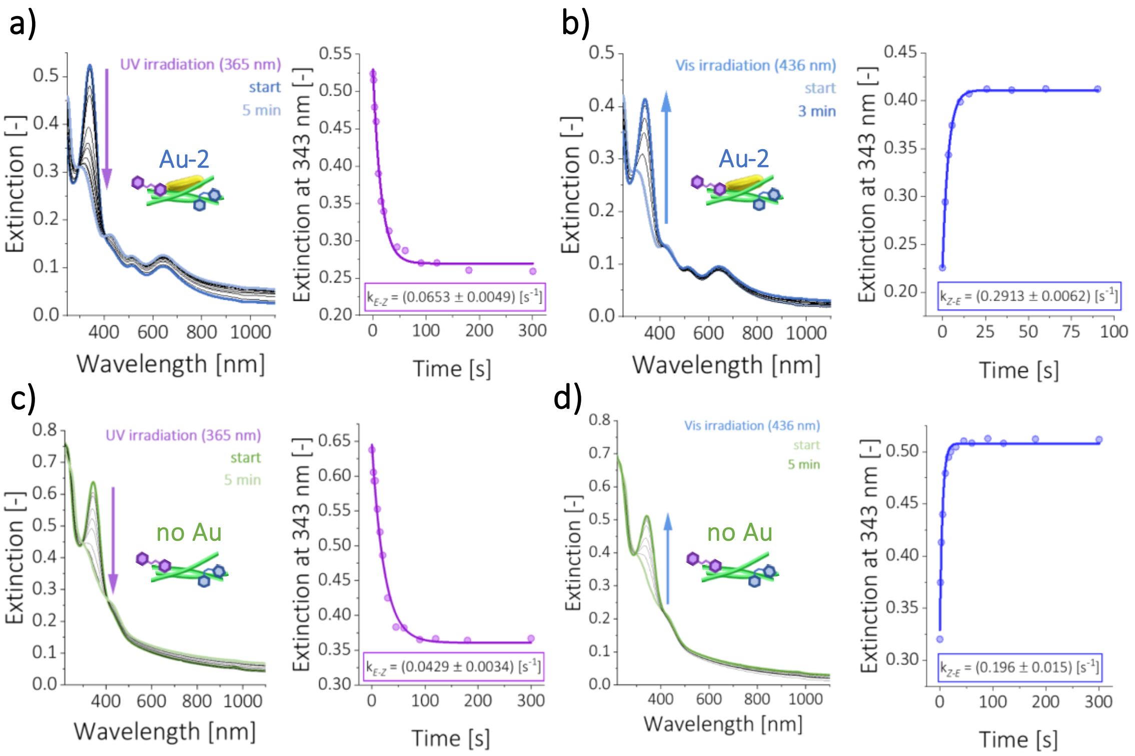

Figure S15. *Spectral profiles and extinction changes as a function of time during isomerization reactions occurring in the hybrid materials.* **a)** *<u>E-Z</u> and* **b)** *<u>Z-E</u> isomerization kinetics for sample containing* ***Au-2*** *plasmonic core (**AzoGly-Au-2-CNFs**).* **c)** *<u>E-Z</u> and* **d)** *<u>Z-E</u> isomerization kinetics for control sample without gold -* ***no Au*** *(**AzoGly-CNFs**).*

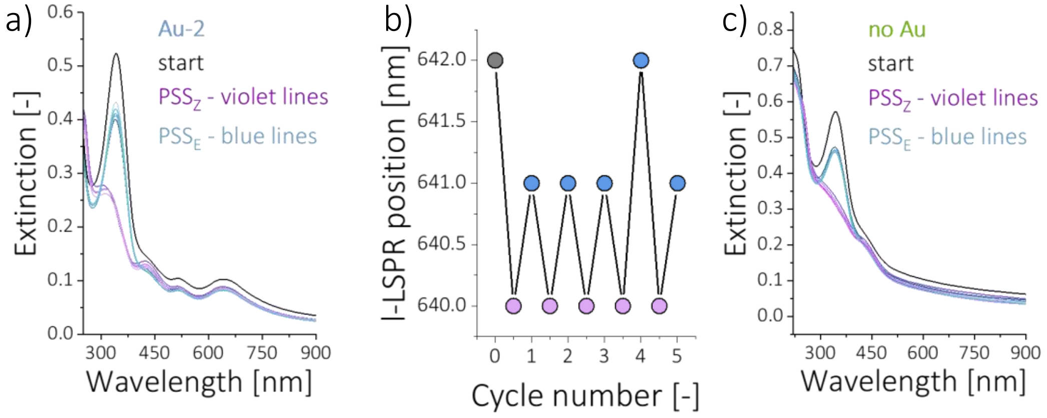

Figure S16. *Changes of the extinction spectra during photostability tests. Reference data for Figure 4c in the main text.* **a)** *Spectral profile changes for sample containing* ***Au-2*** *plasmonic core (**AzoGly-Au-2-CNFs**) accompanied by* **b)** *the evolution of l-LSPR band position in the respective PSSs (purple points indicate PSS_Z and blue points PSS_E).* **c)** *Spectral profile changes for sample without gold -* ***no Au*** *(**AzoGly-CNFs**).*

4.3. Thermal relaxations (*Z-E* isomerization in dark conditions)

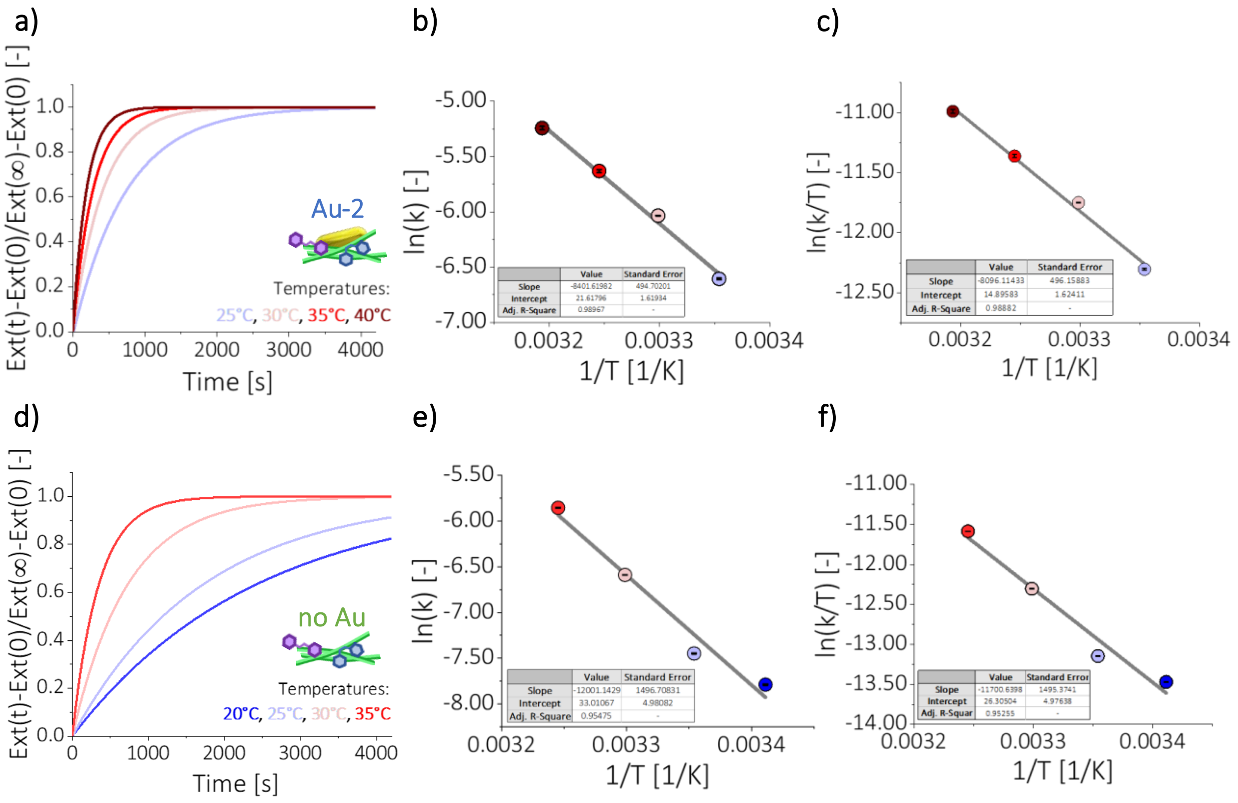

Figure S17. a, d) *Hybrid materials - thermal Z-E AzoGly isomerization in the dark (**Au-2** – top, **no Au** – bottom), accompanied by the appropriate* **b, e)** *Arrhenius and* **c, f)** *Eyring plots. Thermal Z-E isomerizations exhibit first order kinetics.*

Thermodynamic and kinetic parameters of the *Z-E* isomerization in the dark were determined based on Arrhenius (**Equation S2**) and Eyring (**Equation S3**) equations:

Equation S2 $$k_{Z-E} = Ae^{-\frac{E_a}{RT}}$$

Equation S3 $$k_{Z-E} = \frac{k_B T}{h} e^{-\frac{\Delta G^{\ddagger}}{RT}} = \frac{k_B T}{h} e^{\frac{\Delta S^{\ddagger}}{R}} e^{-\frac{\Delta H^{\ddagger}}{RT}}$$

where $k_{Z\text{-}E}$ [s^{-1}] is thermal *Z-E* isomerization rate constant at 298 K and can be recalculated into thermal half-life, $\tau_{1/2}$ [s] ($\tau_{1/2} = \frac{ln2}{k}$); A [s^{-1}] is Arrhenius prefactor; E_a [kJ mol^{-1}] is activation energy; R [J mol^{-1} K^{-1}] is universal gas constant; T [K] is temperature; k_B [J K^{-1}] is Boltzmann constant; h [J·s] is Planck's constant; $\Delta G^{\ddagger}$ [kJ mol^{-1}] is activation free energy; $\Delta S^{\ddagger}$ [J mol^{-1} K^{-1}] is activation entropy, and $\Delta H^{\ddagger}$ [kJ mol^{-1}] is activation enthalpy.

Table S2. *Comparison of the calculated kinetic and thermodynamic parameters for hybrid materials with and without Au.*

Parameter	Sample	
	no Au	Au-2
ln(k$_{Z\text{-}E}$) [-] [a)]	-7.24 ± 7.10	-6.56 ± 2.40
k$_{Z\text{-}E}$ [s^{-1}] [a)]	7.16·10^{-4}	1.41·10^{-3}
τ$_{1/2}$ [min] [a)]	16.13	8.17
A [s^{-1}] ·10^{-11}	2200 ± 2200	0.024 ± 0.020
E$_a$ [kJ mol^{-1}]	99.8 ± 12.5	69.9 ± 4.2
ΔG‡ [kJ mol^{-1}]	91.0 ± 18.1	89.3 ± 6.2
ΔS‡ [J mol^{-1} K^{-1}]	21 ± 42	-74 ± 15
ΔH‡ [kJ mol^{-1}]	97.3 ± 12.5	67.3 ± 4.2

[a)] Estimated from Arrhenius equation at 298 K.

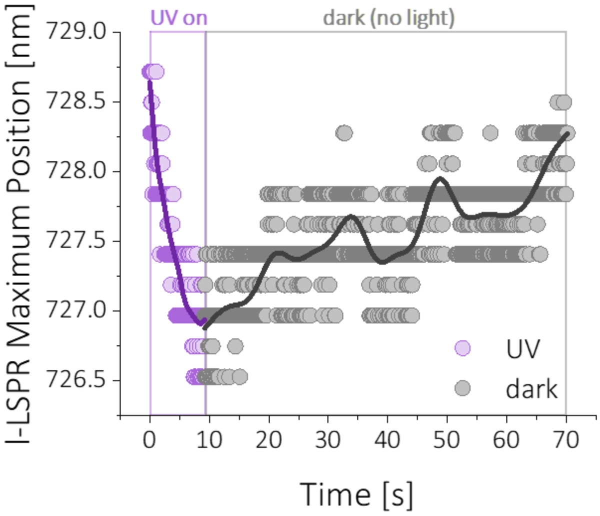

Figure S18. *Changes in the position of the maximum of the l-LSPR band of Au-1 nanorods upon Azo UV-induced (E-Z) and thermal (Z-E, in the dark) isomerization in the hybrid material.*

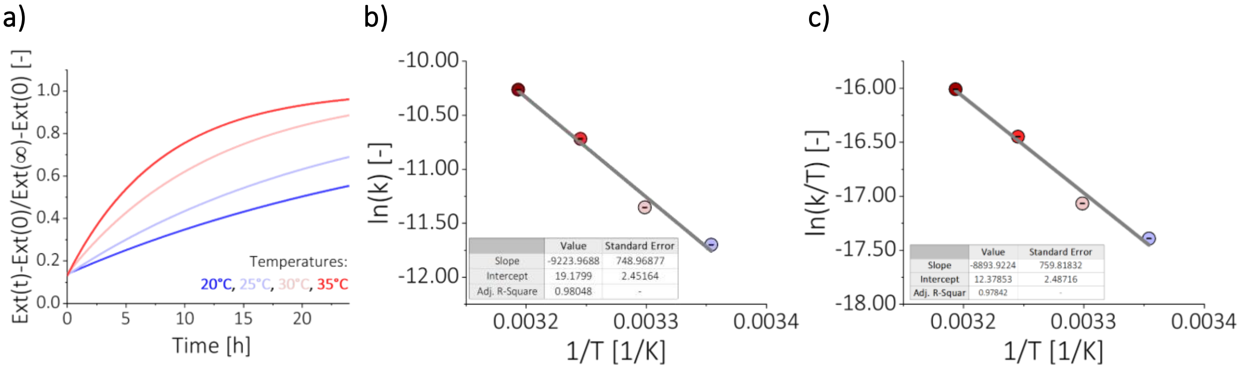

Figure S19. a) *Thermal Z-E AzoGly isomerization for free molecules in EtOH, accompanied by the appropriate* **b)** *Arrhenius and* **c)** *Eyring plots.*

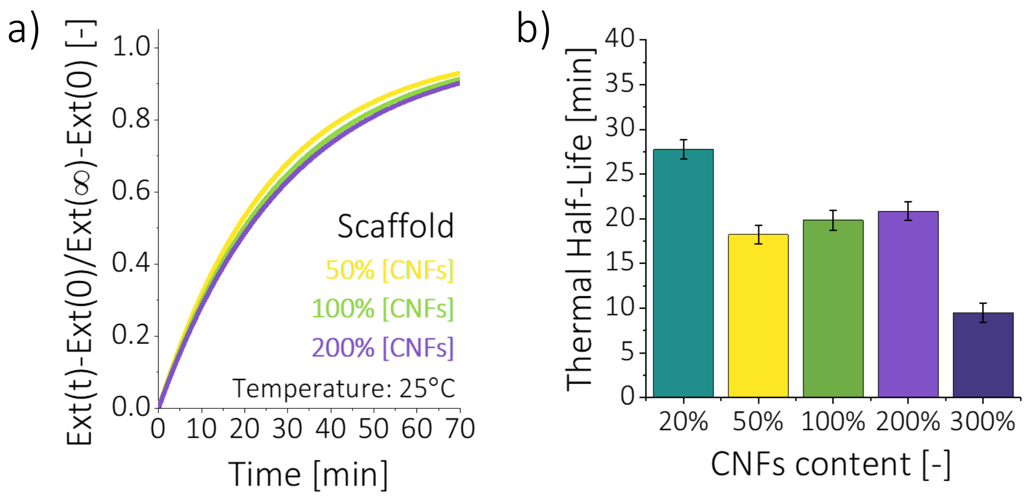

Figure S20. *AzoGly-CNFs samples without gold, containing different amounts of CNFs.* **a)** *For changes in CNFs content between 50% and 200% of CNFs the isomerization of AzoGly in the hybrid fibers is kinetically comparable.* **b)** *The overall comparison of the thermal half-lives.*

Section 5. Plasmon-assisted isomerization of AzoGly

We carried out two types of experiments, (1) with free Azo molecules and (2) Azo-impregnated CNFs. All spectra were acquired in real time every 1 second. We limited the time-frame of the experiment to only 1 hour, to minimize the possibility of material impregnation with AzoGly in scenario (1). Collected data were processed using a custom-written application (R programming language).

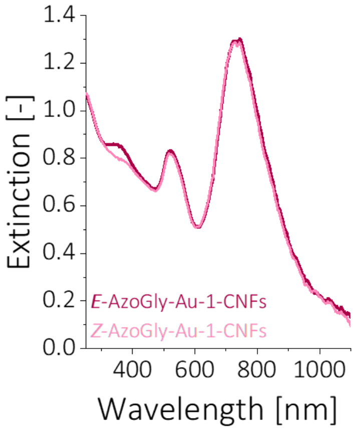

Figure S21. *Extinction spectrum of* ***AzoGly-Au-1-CNFs*** *material. The $\pi \rightarrow \pi^*$ band of AzoGly is overlapped by extinction of Au-1-CNFs framework, and due to the heavy loading of CNFs with AuNRs, the resolution of the measurement (the extent of the $\pi \rightarrow \pi^*$ band intensity change) is decreased.*

5.1. Catalytic influence of AuNRs on free AzoGly (in EtOH)

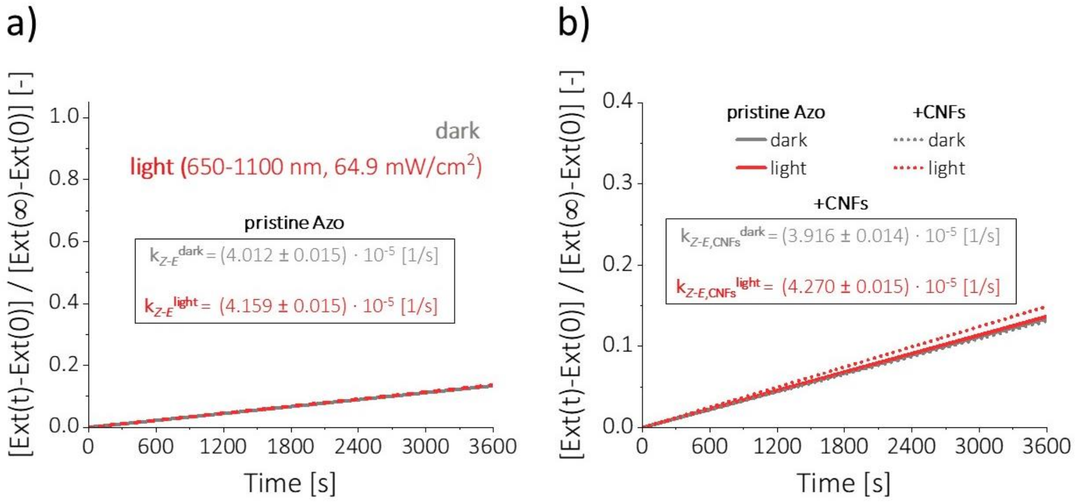

Figure S22. *Azo Z-E isomerizations at room temperature, 25°C.* **a)** *Control irradiation of free AzoGly in EtOH (red line) vs. thermal back-isomerization in the dark (grey line). Difference is negligible.* **b)** *Comparison of the dark vs. light back-isomerizations between pristine AzoGly and AzoGly in the presence of bare CNFs. No catalytic effect of the cellulose scaffold is manifested.*

Relative errors between each pair of the experiments:

- dark (pristine vs. bare CNFs): 2.27%,
- light (pristine, vs. bare CNFs): 2.61%,
- pristine AzoGly (dark vs. light): 3.68%
- addition of bare CNFs (dark vs. light): 8.56%,

are in accordance with acceptable values reported by Gille *et al.*[25]

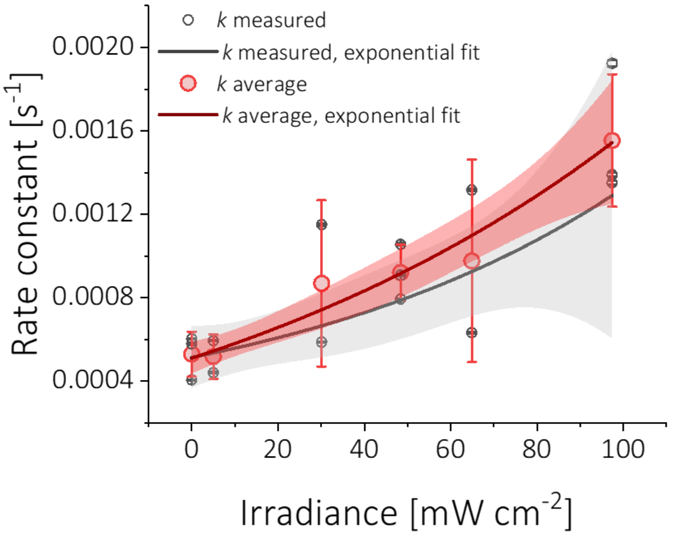

Figure S23. *Rate of Azo isomerization in the hybrid material as a function of light irradiance fitted using exponential function (according to the Arrhenius law) indicates the dominant role of the thermoplasmonic effect.*

Table S3. *The analysis of advantages and disadvantages of the proposed idea of Azo compounds as molecular thermometers juxtaposed with the known nanothermometers.*

Number	Comparison with	Disadvantage
**1**	lanthanide-doped upconverting nanoparticles (doi.org/10.1016/j.cej.2016.03.149)	Azo molecular thermometers need careful calibration of their photoswitching under chosen conditions, unlike the lanthanide-doped upconverting nanoparticles, which rely on the fluorescence intensity ratio technique characterized by reduced influence of different factors and measuring conditions on the detection. Such requirement is, however, not rare for molecular and nanoscale sensors.
**2**	DNA nanothermometers (doi.org/10.1021/acs.nanolett.6b00156)	'On the spot' reprogramming of the detection range for certain Azo molecules may be difficult, unlike for DNA nanothermometers. Nevertheless, Azo compounds are prone to the indirect control over their photoswitching, hence, it is conceivable that the addition of appropriate chemical species can mitigate such disadvantage.
**3**	Zn-based metal-organic frameworks (doi.org/10.1038/ncomms15985)	Many Azo compounds, due to their hydrophobic nature, cannot be readily used in water-based systems, unlike Zn-based metal-organic frameworks. Nevertheless, this disadvantage can be mitigated by proper system design, just as we presented in this work, where photochromic Azo component is water-functional despite its initial insolubility in water.
**4**	overall	Azo compounds can possibly decompose at higher temperatures, although the theoretical detection range based on the thermal stability of Azo molecules (which is typically around 473-523 K[26-27], but can be also extended up to 623 K[28] or even 803 K[29]) is comparable with other nanothermometers.
**Number**	**Comparison with**	**Advantage**
**1**	DNA nanothermometers (doi.org/10.1021/acs.nanolett.6b00156),	Single Azo photoswitch can be used in different systems, setups, and experimental scenarios, similarly to DNA nanothermometers.
**2**	Raman luminescent nanothermometers (doi.org/10.1002/adom.202201675)	Azo kinetic changes can be monitored using simple and inexpensive technique, such as UV-Vis absorption spectroscopy, unlike Raman luminescent nanothermometers.
**3**	single-molecule magnets (doi.org/10.1002/anie.202306970)	Similarly to single-molecule magnets the proposed methodology relies on the sensitivity of the inherent features of Azo compounds towards temperature changes. Moreover, Azo compounds are examples of single-molecule based materials and, hence, can be integrated in the technologies of the future.
**4**	overall	The vast library of available Azo compounds, differing in e.g. solubility, grafting potential, and thermal stability, makes them a ready-to-use class of temperature sensors for various systems and applications.

5.2. Plasmon-assisted isomerization of AzoGly in the hybrid material - ARIMA model

The ARIMA model consists of four basic components: autoregressive (AR) model, moving average (MA) model, seasonal model, and differencing (integration).[6] The seasonal part of ARIMA approach does not apply to our data. A non-seasonal ARIMA model is described by (p, d, q) basic notation. p, d, q are positive integers, and can be defined as follows:

1. p – the order of the AR part of the model
2. d – the degree of non-seasonal differencing
3. q – the order of the MA part of the model.

AR, MA and differencing components of the model allow us to properly model the temporal serial structure of observations. Additionally, covariates enable modelling time series with additional independent variables. Schaffer *et al.*[6] described three particular covariates that can be used to model the effect of intervention on time series data. We expect to see only one of them, namely a Ramp-type change (change in slope) that occurs in our time series immediately after the intervention (when light is switched on). The change in slope of the data corresponds to change in the AzoGly isomerization rate upon triggering thermoplasmonic effect. Thus, we model the linearized extinction value Y_t as a function of previous values of Y (AR model), previous values of the error term (MA model), differencing, and a Ramp variable. Let us note that while this is a linear model, predicted values will not lie on the same line due to dependence on a varying set of past values.

ARIMA models require specifications of numbers of lagged values of Y, ϵ, and differences that will be used. To select these values, we used the automated model selection approach implemented in the auto.arima function of forecast package[7] available in the R statistical environment[8]. Thus, an ARIMA model is fitted separately for each pair of dark and light (OFF-ON) time periods for each sample. As a result we obtain quantitative measures of Ramp effect (**Table S6**). For each model we test statistical significance of Ramp effect by calculating p-values (**Table S7**). Significant result of the test suggests that there is a post-intervention difference in slope (rate of the extinction change) and that difference between dark and light segments cannot be attributed to random variation of the data. We account for multiple comparisons in each sample by applying the Benjamini-Hochberg correction.[9]

Table S4. *Description of the investigated samples. Hybrid materials either contained AuNRs (investigated samples – 1 and 2) or not (control samples – 3 and 4). The same irradiation conditions were applied. Two variants of experiments were performed – with different time intervals (2.5 or 5.0 min).*

Sample ID	Au	Spectral range [nm]	Power density [mW/cm^2]	Time interval [min]
**1**	YES	650-1100	145.8	5.0
**2**	YES			2.5
**3**	NO			5.0
**4**	NO			2.5

Table S5. *Selected values of p, d, and q parameters (for ARIMA) for the considered intervention – switching the light on.*

Sample	OFF-ON pair	p (AR order)	d (degree of differencing)	q (MA order)
**1**	1	1	1	1
	2	1	1	2
	3	2	0	1
	4	3	1	2
	5	0	1	2
	6	1	1	4
**2**	1	2	1	2
	2	1	1	1
	3	2	1	2
	4	1	1	1
	5	1	0	1
**3**	1	1	2	0
	2	4	2	0
	3	0	1	1
	4	0	1	1
	5	0	1	2
	6	5	1	0
**4**	1	1	2	0
	2	0	1	1
	3	1	1	1
	4	0	1	1
	5	1	1	1

Table S6. *Estimated values of the Ramp effect for each sample and OFF-ON pair within a sample for the considered intervention – switching the light on.*

Sample	Content	Pair 1	Pair 2	Pair 3	Pair 4	Pair 5
**1**	Au	-0.00019	-0.00074	-0.00056	-0.00025	0.00119
**2**	Au	-0.00207	-0.00160	-0.00146	-0.00275	-
**3**	no Au	-0.00502	-0.00301	-0.00015	0.00045	-0.00178
**4**	no Au	0.01356	-0.00015	-0.00066	0.00000	-0.00403

Table S7. *Adjusted p-values for tests of significance of the Ramp effect for each sample and OFF-ON pair within a sample for the considered intervention – switching the light on. Green colour indicates statistically significant differences. Red colour indicates lack of the difference. Orange colour indicates values relatively close to the p=0.05 level. Values marked with asterisk are not equal before performing Benjamini-Hochberg correction. Their values before correction are given in the brackets.*

Sample	Content	Pair 1	Pair 2	Pair 3	Pair 4	Pair 5
**1**	Au	0.00028	0.00000	0.00000	0.12451	0.01207
**2**	Au	0.00000	0.00073* (0.00073)	0.00073* (0.00052)	0.00073* (0.00067)	-
**3**	no Au	0.82276* (0.63962)	0.82276* (0.76667)	0.82276* (0.58053)	0.82276* (0.55923)	0.23613
**4**	no Au	0.38182	0.38182	0.07253	0.99606	0.06671

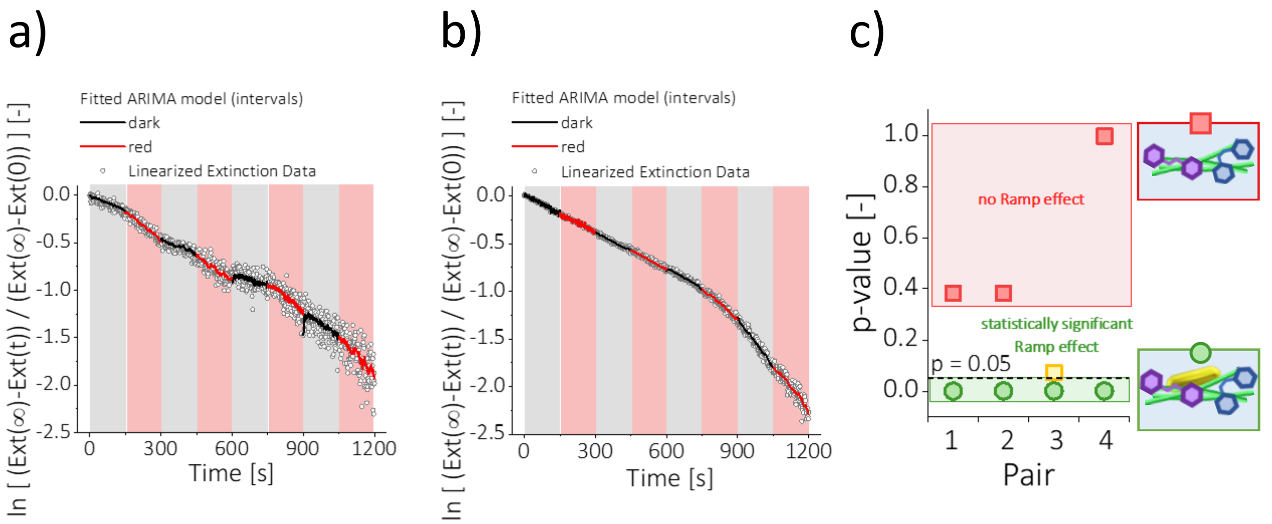

Figure S24. *Results of the statistical modelling (ARIMA, OFF-ON pairs, intervention: light on) for* **a)** *sample 2 (with gold) and* **b)** *sample 4 (without gold).* **c)** *Graphical summary of the significance of the estimated Ramp effect values, indicating that only in the presence of Au the selected irradiation conditions induce changes in the AzoGly isomerization in the hybrid material.*

For sample **4**, for the third OFF-ON pair, small disruption of the linear trend occurs. We assign it to material's sedimentation, which, although corrected during data treatment, may still affect measurements to some degree. This emphasizes importance of the OFF-ON experiments and proves that their results can be analyzed impartially, based on the objective, quantitative measures.

Below we present results of the modelling (ARIMA model) considering switching the light off as an intervention.

Table S8. *Selected values of p, d, and q parameters (for ARIMA) for the considered intervention – switching the light off.*

Sample	ON-OFF pair	p (AR order)	d (degree of differencing)	q (MA order)
**1**	1	0	1	2
	2	0	1	1
	3	0	1	2
	4	2	1	2
	5	0	1	2
	6	0	1	2
**2**	1	0	1	1
	2	0	1	1
	3	1	1	1
	4	0	1	1

**3**	1	2	2	2
	2	0	1	1
	3	0	1	1
	4	1	1	1
	5	2	1	4
	6	4	1	0
**4**	1	0	1	1
	2	0	1	1
	3	1	1	2
	4	2	1	1
	5	1	1	0

Table S9. *Estimated values of the Ramp effect for each sample and ON-OFF pair within a sample for the considered intervention – switching the light off.*

Sample	Content	Pair 1	Pair 2	Pair 3	Pair 4	Pair 5
**1**	Au	0.00045	0.00108	0.00014	-0.00080	-0.00117
**2**	Au	-0.00125	0.00090	-0.00183	-0.00215	-
**3**	no Au	0.00001	0.00005	0.00005	-0.00003	0.00007
**4**	no Au	-0.00002	-0.00008	-0.00117	-0.00115	-0.26463

Table S10. *Adjusted p-values for tests of significance of the Ramp effect for each sample and ON-OFF pair within a sample for the considered intervention – switching the light off. Green colour indicates statistically significant differences. Red colour indicates lack of difference. Orange colour indicates values relatively close to the p=0.05 level. Values marked with asterisk are not equal before performing Benjamini-Hochberg correction. Their values before correction are given in the brackets.*

Sample	Content	Pair 1	Pair 2	Pair 3	Pair 4	Pair 5
**1**	Au	0.00000	0.00000	0.13287	0.03655	0.00005
**2**	Au	0.06823*<br>(0.04048)	0.06823*<br>(0.05117)	0.06823*<br>(0.0209)	0.09004	-
**3**	no Au	0.98750*<br>(0.98750)	0.98750*<br>(0.75944)	0.98750*<br>(0.93775)	0.98750*<br>(0.95901)	0.98750*<br>(0.97923)
**4**	no Au	0.86521	0.80271	0.00000	0.34725	0.80271

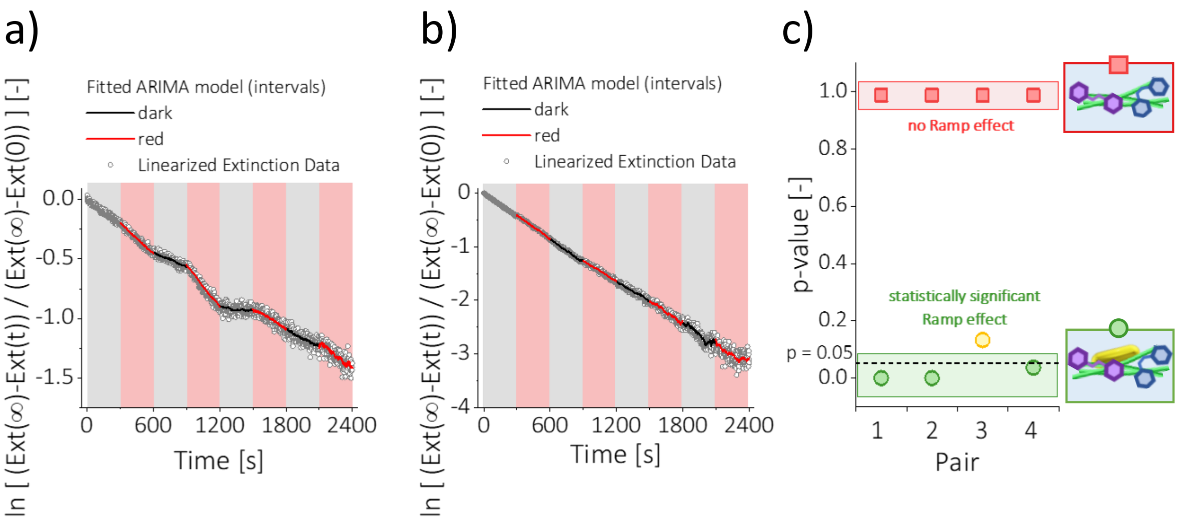

Figure S25. *Results of the statistical modelling (ARIMA, ON-OFF pairs, intervention: light off) for* **a)** *sample 1 (with gold) and* **b)** *sample 3 (without gold).* **c)** *Graphical summary of the significance of the estimated Ramp effect values, indicating that only in the presence of Au upon turning the light off after long irradiation intervals changes in the AzoGly isomerization in the hybrid material occur.*

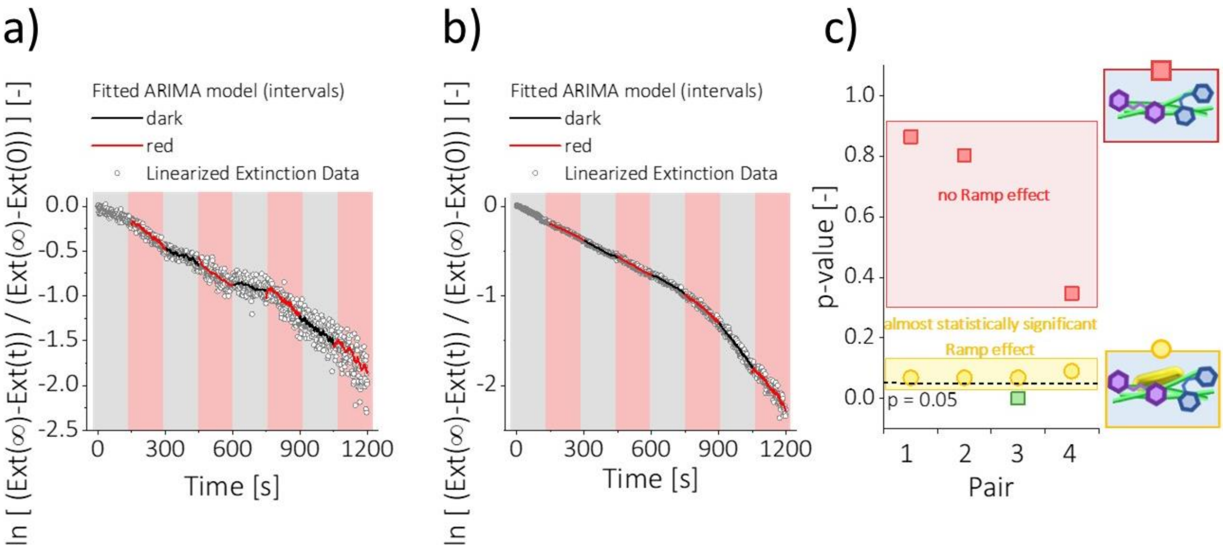

Figure S26. *Results of the statistical modelling (ARIMA, ON-OFF pairs, intervention: light off) for* **a)** *sample 2 (with gold) and* **b)** *sample 4 (without gold).* **c)** *Graphical summary of the significance of the estimated Ramp effect values, indicating that in the presence of Au upon turning the light off after short irradiation intervals changes in the AzoGly isomerization in the hybrid material occur, however, they are not as pronounced as for longer irradiation intervals.*

References

[1] H. H. Chang, C. J. Murphy, *Chemistry of Materials* **2018**, *30*, 1427.
[2] L. Vigderman, E. R. Zubarev, *Chemistry of Materials* **2013**, *25*, 1450.
[3] B. Nikoobakht, M. A. El-Sayed, *Chemistry of Materials* **2003**, *15*, 1957.
[4] M. Liu, P. Guyot-Sionnest, *Journal of Physical Chemistry B* **2005**, *109*, 22192.
[5] N. Tarnowicz-Staniak, S. Vázquez-Díaz, V. Pavlov, K. Matczyszyn, M. Grzelczak, *ACS Appl Mater Interfaces* **2020**, *12*, 19377.
[6] A. L. Schaffer, T. A. Dobbins, S. A. Pearson, *BMC Med Res Methodol* **2021**, *21*, 1.
[7] R. J. Hyndman, Y. Khandakar, *J Stat Softw* **2008**, *27*, 1.
[8] R Core Team, **2022**.
[9] Y. Benjamini, Y. Hochberg, *Journal of the Royal Statistical Society. Series B (Methodological)* **1995**, *57*, 289.
[10] H. M. D. Bandara, S. C. Burdette, *Chem Soc Rev* **2012**, *41*, 1809.
[11] P. Rao, R. Doremus, *J Non Cryst Solids* **1996**, *203*, 202.
[12] T. Hendel, M. Wuithschick, F. Kettemann, A. Birnbaum, K. Rademann, J. Polte, *Anal Chem* **2014**, *86*, 11115.
[13] J. Rodríguez-Fernández, J. Pérez-Juste, P. Mulvaney, L. M. Liz-Marzán, *Journal of Physical Chemistry B* **2005**, *109*, 14257.
[14] L. Scarabelli, M. Grzelczak, L. M. Liz-Marzán, *Chemistry of Materials* **2013**, *25*, 4232.
[15] N. G. Khlebtsov, B. N. Khlebtsov, E. V. Kryuchkova, S. V. Zarkov, A. M. Burov, *The Journal of Physical Chemistry C* **2022**, 19268.
[16] R. Huang, Y. H. Wen, G. F. Shao, Z. Z. Zhu, S. G. Sun, *RSC Adv* **2014**, *4*, 7528.
[17] T. K. Sau, C. J. Murphy, *Langmuir* **2004**, *20*, 6414.
[18] E. Carbó-Argibay, B. Rodríguez-González, S. Gómez-Graña, A. Guerrero-Martínez, I. Pastoriza-Santos, J. Pérez-Juste, L. M. Liz-Marzán, *Angewandte Chemie International Edition* **2010**, *49*, 9397.
[19] J. Zhang, J. K. Whitesell, M. A. Fox, *Chemistry of Materials* **2001**, *13*, 2323.
[20] R. Klajn, P. J. Wesson, K. J. M. Bishop, B. A. Grzybowski, *Angewandte Chemie International Edition* **2009**, *48*, 7035.
[21] K. H. Shin, J. S. Eun, *Bull Korean Chem Soc* **2008**, *29*, 1259.
[22] T. Weidner, F. Bretthauer, N. Ballav, H. Motschmann, H. Orendi, C. Bruhn, U. Siemeling, M. Zharnikov, *Langmuir* **2008**, *24*, 11691.
[23] U. Siemeling, C. Bruhn, F. Bretthauer, M. Borg, F. Träger, F. Vogel, W. Azzam, M. Badin, T. Strunskus, C. Wöll, *Dalton Transactions* **2009**, 8593.
[24] R. Klajn, *Pure and Applied Chemistry* **2010**, *82*, 2247.
[25] K. Gille, H. Knoll, K. Quitzsch, *Int J Chem Kinet* **1999**, *31*, 337.
[26] T. L. Nguyen, M. A. Saleh, *Results in Chemistry* **2020**, 2, 100085.
[27] M. Kazem-Rostami, *J Therm Anal Calorim* **2020**, 140, 613.
[28] H. Chi, K. Y. Mya, T. Lin, C. He, F. Wang, W. S. Chin, *New J. Chem.* **2013**, 37, 735.
[29] S. Guo, Y. Zhang, R. Xiong, A. K. Singh, *Case Stud. Therm. Eng.* **2023**, 52, 103774.
